# Identification of serum miR-1246 and miR-150-5p as novel diagnostic biomarkers for high-grade serous ovarian cancer

**DOI:** 10.1038/s41598-023-45317-7

**Published:** 2023-11-07

**Authors:** Magdalena Niemira, Anna Erol, Agnieszka Bielska, Anna Zeller, Anna Skwarska, Karolina Chwialkowska, Mariusz Kuzmicki, Jacek Szamatowicz, Joanna Reszec, Pawel Knapp, Marcin Moniuszko, Adam Kretowski

**Affiliations:** 1 Clinical Research Centre, Medical University of Bialystok, Bialystok, Poland; 2 Cancer Center, Department of Oncology, Albert Einstein College of Medicine, Bronx, NY USA; 3 Centre for Bioinformatics and Data Analysis, Medical University of Bialystok, Bialystok, Poland; 4 Department of Gynecology and Gynecological Oncology, Medical University of Bialystok, Bialystok, Poland; 5 Department of Medical Pathomorphology, Medical University of Bialystok, Bialystok, Poland; 6 University Oncology Centre, University Clinical Hospital in Bialystok, Bialystok, Poland; 7 Department of Regenerative Medicine and Immune Regulation, Medical University of Bialystok, Bialystok, Poland

**Keywords:** Gynaecological cancer, Diagnostic markers

## Abstract

Epithelial ovarian cancer (EOC) is one of the leading cancers in women, with high-grade serous ovarian cancer (HGSOC) being the most common and lethal subtype of this disease. A vast majority of HGSOC are diagnosed at the late stage of the disease when the treatment and total recovery chances are low. Thus, there is an urgent need for novel, more sensitive and specific methods for early and routine HGSOC clinical diagnosis. In this study, we performed miRNA expression profiling using the NanoString miRNA assay in 34 serum samples from patients with HGSOC and 36 healthy women. We identified 13 miRNAs that were differentially expressed (DE). For additional exploration of expression patterns correlated with HGSOC, we performed weighted gene co-expression network analysis (WGCNA). As a result, we showed that the module most correlated with tumour size, nodule and metastasis contained 8 DE miRNAs. The panel including miR-1246 and miR-150-5p was identified as a signature that could discriminate HGSOC patients with AUCs of 0.98 and 1 for the training and test sets, respectively. Furthermore, the above two-miRNA panel had an AUC = 0.946 in the verification cohorts of RT-qPCR data and an AUC = 0.895 using external data from the GEO public database. Thus, the model we developed has the potential to markedly improve the diagnosis of ovarian cancer.

## Introduction

Epithelial ovarian cancer (EOC) is diagnosed in an estimated 314,000 women, accounting for 210,000 deaths worldwide annually (GLOBOCAN 2020; https://doi.org/10.3322/caac.21660). In 2022, in the United States, over 12,000 women died because of ovarian cancer, including over 9000 at postmenopausal age^1^. High-grade serous ovarian cancer (HGSOC) is EOC’s most common histological subtype, with a five-year survival rate below 30%. While the disease limited only to the ovaries (stage I) can be cured in up to 90% of patients, most cases are diagnosed at a late stage (stage III or IV)^2^. The routine diagnostic procedures, including transvaginal ultrasonography and CA125 antigen measurement, are unsuitable for early diagnosis^3,4^. Several protein-based biomarkers, such as CA125 and HE4, are currently being used for FDA-approved diagnosis. The most studied marker, CA125, is only elevated by 50–60% of early-stage EOC or asymptomatic patients. Recent findings suggest that CA125 allows the diagnosis of EOC with 88.7% sensitivity and 74.7% specificity^5–7^. Combined detection of CA125 and human epididymis secretory protein E4 (HE4) has been shown to improve EOC screening^8–10^. For example, the Risk of Ovarian Malignancy Algorithm (ROMA) with CA125 and HE4 as biomarkers, showed 100% sensitivity and 74.2% specificity in preoperative assessment^11,12^. Rein et al.^13^ showed that Ova1 panel testing upregulated CA125-II and ß2M and downregulated ApoA1, transthyretin, and transferrin, allowing for EOC diagnosis in 96% of cases with 96% sensitivity and 35% specificity. Given the lack of usual symptoms of EOC and the limitations in early diagnosis identifying more sensitive and specific biomarkers is urgently required.

Several groups recently showed the pivotal role of microRNAs (miRNAs) as potential biomarkers for various cancers. Circulating miRNA has many features to fulfil the role of a useful biomarker. They are highly stable and resistant to RNase digestion, high temperatures, extreme pH, extended storage, and multiple freeze–thaw cycles^14^. MiRNAs are short (~ 22 nucleotides), non-coding, single-stranded RNA molecules that play a crucial role in regulating gene expression by interacting with the 3’-UTR region of mRNAs^15,16^. Since a single miRNA can interact with many mRNAs, miRNAs regulate several cellular processes, such as differentiation, development, proliferation, metabolism, tumorigenesis, apoptosis and angiogenesis^17–19^. In addition, given the nature of miRNA-regulated genes, these molecules can act as oncogenes (oncomiRs) and tumour suppressors^20^. However, the current literature often depicts the contradictory role of particular miRNAs in cancer development. It has been hypothesised that such discrepancies may result from the ability of miRNAs to simultaneously affect the expression of both tumour-suppressive and oncogenic mRNAs. For instance, miR-186 may serve as an oncomiR or a tumour suppressor miRNA. Its dual role is related to enhancing or inhibiting proliferation, metastasis, invasion, and apoptosis in cancers^21^. Similarly, while miR-155 was initially identified as an oncomiR in pancreatic cancer^22^, in ovarian and gastric cancers, its expression is inhibited, indicating that miR-155 may also act as a tumour suppressor^23^.

Studies on epithelial ovarian cancer have shown dysregulation of several miRNAs, such as miR-200a, miR-200b, miR-200c and miR-199a in cancer tissue^24^, and circulating miRNAs in blood, including miR-130-3p, miR-143-3p, miR-145, miR-200c, miR-205-5p, miR-26a-5p, miR-328-3p, miR-374a-5p, and miR-766-3p^25,26^. Importantly, while numerous studies have already demonstrated the diagnostic or the prognostic potential of circulating miRNAs in ovarian cancer^27–29^, most of these analyses were performed bulking different subtypes of ovarian cancer regardless of histology. It is worth underlining that different histological subtypes may contribute to contradictory alterations of miRNAs in specific cancers. For example, Schmid et al.^30^ analysed miR-34a expression in EOC and found that miR-34a was significantly downregulated in serous, endometroid and mucinous histological subtypes, while no significant alteration in clear cell ovarian cancer was found in comparison with healthy ovary tissues.

Despite many reports, no decisive circulating miRNA signature has been proposed to distinguish HGSOC patients from healthy individuals unambiguously. Herein, we propose that simultaneous identification of miR-1246 and miR-150-5p in serum can be used for the non-invasive diagnosis of HGSOC patients. We also investigated the possible involvement of circulating miRNAs in HGSOC development by analysing the biological importance of miRNA targets and the functional enrichment profile of their target gene sets.

## Results

### Identification and validation of differentially expressed miRNAs in serum samples of HGSOC patients

**Figure 1:**
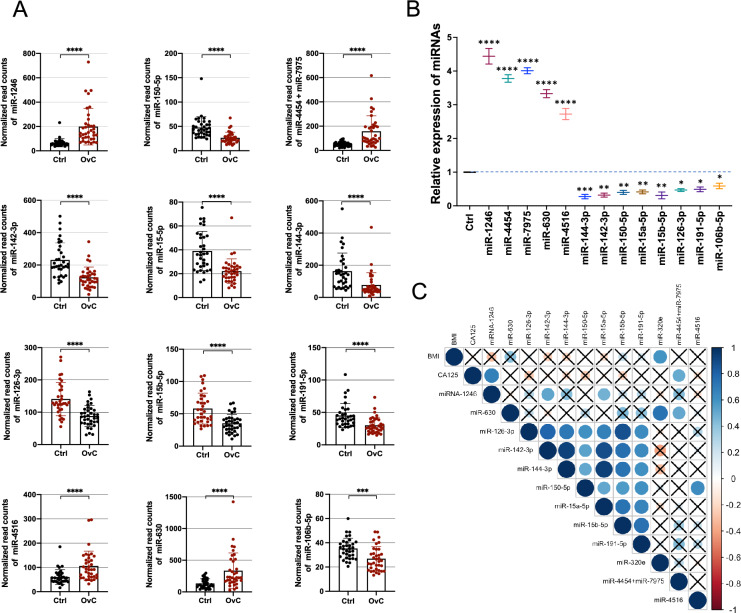
Differential miRNAs expression in the serum of HGSOC patients and healthy control. (**A**) The scatter plot with the bar shows the expression levels of circulating miRNAs in serum in HGSOC compared to healthy control from the NanoString platform. Differences in the expression levels in miRNAs between patients and controls were compared using the Mann–Whitney test; (**B**) RT-qPCR validation of 13 miRNAs selected from the NanoString platform. Each bar represents the mean ratio of differentially expressed (DE) miRNAs expression and miR-103a-3p and miR-199b-5p as reference miRNAs ± standard error of the mean (SEM). Asterisks indicate a significant difference compared to the control (****p < 0.0001, ***p < 0.001, **p < 0.01, *p < 0.05); (**C**) Pearson correlation results of the DE miRNAs and BMI and CA125 levels. The cross stays for not significant results.

Altogether, 13 out of 798 unique miRNAs showed significant differences in counts between HGSOC and healthy donor serum samples (Table 1) with Fold Change (FC) >|1.5| and False Discovery Rates (FDRs) < 0.05 (Fig. 1A). Table 2 summarises FCs and FDRs for all thirteen differentially expressed (DE) miRNAs. Five miRNAs were upregulated: miR-1246, miR-4454 + miR-7975 (the mature sequence of miR-7975 differs only by one base from miR-4454), miR-630, and miR-4516, whereas eight miRNAs were downregulated: miR-144-3p, miR-142-3p, miR-150-5p, miR-15a-5p, miR-15b-5p, miR-126-3p, miR-191-5p, and miR-106b-5p. To validate the diagnostic usefulness of the identified miRNAs, we performed RT-qPCR analysis. All DE miRNAs were detectable by quantitative PCR, and the expression data were comparable to those generated using the NanoString nCounter System (Fig. 1B). Next, we used Pearson tests to calculate the correlation coefficient between the expression of miRNAs and the following patients’ characteristics: body mass index (BMI), and CA125 serum level. We observed a positive correlation between CA125 level and miR-1246 as well as miR-4454 + miR-7975 expression with correlation coefficients of 0.670 and 0.526, respectively y (*P* value = 8.8 × 10^–04^ and *P* value = 1.41 × 10^–02^). In addition, there was also a group of miRNAs, which was correlated with each other, including miR-106b-5p, miR-126-3p, miR-142-3p, miR-144-3p, miR-150-5p, miR-15a-5p, and miR-15b-5p (Fig. 3C).

**Table 1:** Participant characteristics of the NanoString and RT-qPCR model set.

Characteristics	Cases for NanoString analysis						Cases for RT-qPCR validation					
	Training set			Test set			Training set			Test set		
	Cases (n = 28)	Controls (n = 21)	*P* val	Cases (n = 8)	Controls (n = 13)	*P* val	Cases (n = 28)	Controls (n = 34)	*P* val	Cases (n = 14)	Controls (n = 12)	*P* val
Age (mean ± SD)	61.6 ± 14	58.1 ± 6	0.2	59.2 ± 6	61.0 ± 4	0.7	61.3 ± 13	59.3 ± 7	0.3	57.7 ± 13	57.8 ± 7	0.9
BMI (mean ± SD)	29.2 ± 6	27.2 ± 3	0.2	27.9 ± 4	28.7 ± 3	0.4	28.8 ± 5	28.0 ± 3	0.9	28.9 ± 6	26,4 ± 2	0.2
FIGO stages, n (%)												
FIGO I	2 (7.1)	–		1 (12.5)	–		3 (10.8)	–		0 (0.0)	–	
FIGO II	0 (0.0)	–		1 (12.5)	–		0 (0.0)	–		1 (7.1)	–	
FIGO III	15 (53.6)	–		3 (37.5)	–		14 (50.0)	–		6 (42.9)	–	
FIGO IV	10 (35.7)	–		3 (37.5)	–		9 (32.1)	–		5 (35.7)	–	
N/A	1 (3.6)	–		0 (0.0)	–		2 (7.1)	–		2 (14.3)	–

**Table 2:** Overview of the DE miRNAs (FC) >|1.5| in HGSOC concerning normal control.

miRNA	FC	FDR
miR-1246	2.61	< 0.001
miR-150-5p	− 1.81	< 0.001
miR-4454 + miR-7975	2.45	< 0.001
miR-142-3p	− 1.94	< 0.001
miR-191-5p	− 1.51	< 0.001
miR-15b-5p	− 1.65	< 0.001
miR-126-3p	1.61	< 0.001
miR-15a-5p	− 1.75	< 0.001
miR-144-3p	− 2.31	0.01
miR-4516	1.68	0.01
miR-630	2.16	0.01
miR-106b-5p	− 1.56	0.04

*FC* fold change, *FDR* false discovery rate.

### Pathways enrichment analysis of DE miRNA target genes

**Figure 2:**
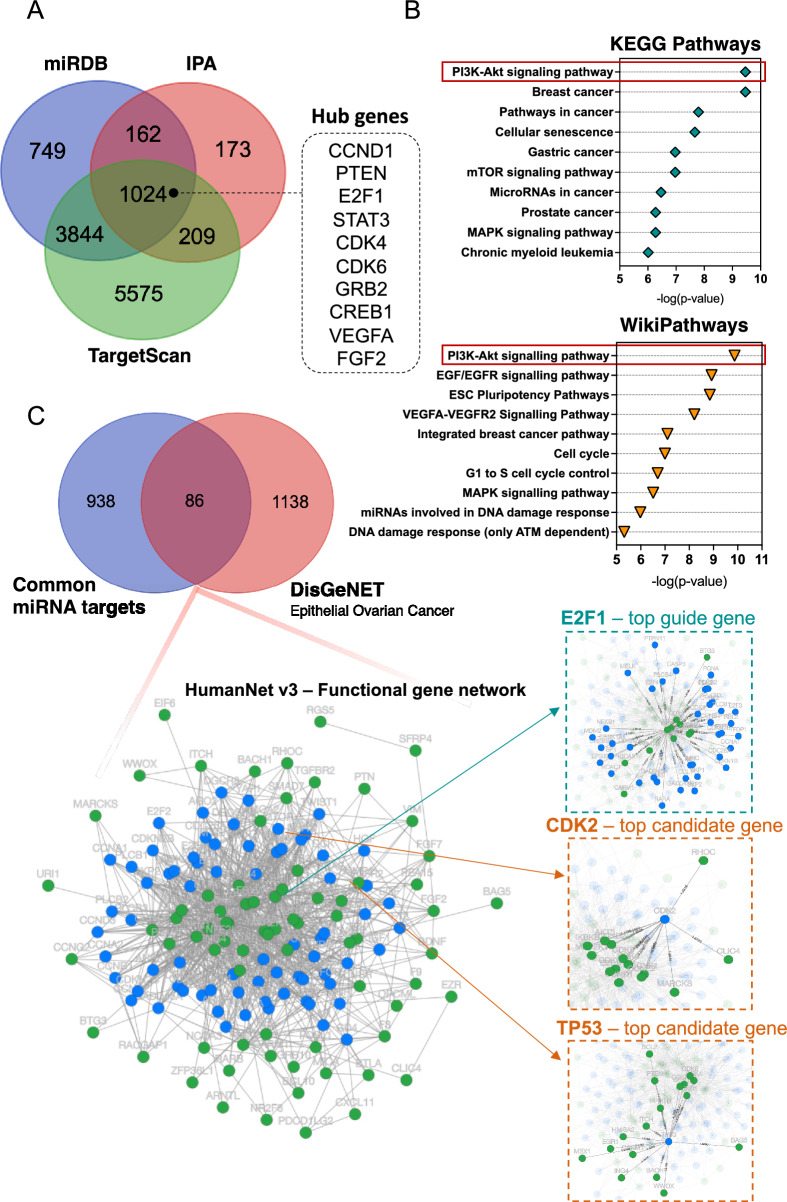
Functional annotation analysis of predicted miRNA-target genes. (**A**) Venn diagram showing the number of miRNA target genes that are shared between different databases, miRDB, TargetScan, and IPA; *miRDB* microRNA target prediction database; *IPA* ingenuity pathway analysis; (**B**) KEGG pathway and WikiPathways analysis of 1024 miRNA target genes. All functional annotations were performed with the hypergeometric test and Bonferroni adjustment (corrected P value (FDR) ≤ 0.05); *KEGG* Kyoto encyclopedia of genes and genomes; (**C**) Venn diagram showing the overlap between 1024 miRNA target genes and genes related to EOC from the DisGeNET database. Additionally, integrated functional gene network analysis of 86 miRNA target genes with the HumanNet v3 platform. Network nodes represent guide (miRNA target genes – green) genes and candidate genes (blue). Edges represent their associations. Edges guide and edges between guide genes and candidate genes are presented; *EOC* epithelial ovarian cancer; *DisGeNET* a database of gene-disease associations.

To reveal the biological function of the DE miRNAs, we first identified 1568 putative DE miRNAs target genes using Ingenuity Pathway Analysis (IPA)^31^**,** 5779 target genes via mirDB^32^ and 10,652 target genes via TagetScanHuman 8.0^33^. For further functional studies, we used 1024 targets that overlapped between the above databases (Fig. 2A). The protein–protein interaction (PPI) network, including 1022 nodes and 4485 edges, was generated by Cytoscape v.3.9.1^34^. Because the network size exceeded 2000 nodes, we reduced the network to proteins that directly interact with each other (Supplementary Fig. S1). For this purpose, using a zero-order network function of the NetworkAnalyst 3.0 platform^35^, we obtained 255 nodes and 497 edges. By ranking the PPI network nodes using eight topological analysis methods, including both local- and global-based algorithms from the cytoHubba plugin^36^ of Cytoscape software, we found that *CCND1*, *PTEN*, *E2F1*, *STAT3*, *CDK4*, *CDK6*, *GRB2*, *RAC1*, *CREB1*, and *VEGFA* scores ranked in the top 10 (Supplementary Table S1). The highly connected hub gene *CCND1* encodes cyclin D1, which together with CDK4 and CDK6 kinases control G1 to S phase cell cycle progression and is frequently overexpressed in human cancers, promoting tumorigenesis^37,38^.

Next, we used a combination of IPA, gene ontology (GO), Kyoto Encyclopedia of Genes and Genomes (KEGG), and WikiPathways^39^ pathway enrichment analyses using the enrichR tool^40^. For biological processes (BP), the DE miRNA target genes were enriched in the regulation of cell proliferation (FDR-corrected *P* value = 8.50 × 10^–09^), G1/S transition of the mitotic cell cycle (FDR-corrected *P* value = 3.44 × 10^–06^), cell cycle G1/S phase transition (FDR-corrected *P* value = 1.84 × 10^–05^), positive regulation of cell population proliferation (FDR-corrected *P* value = 2.71 × 10^–05^), and negative regulation of mitotic cell cycle phase transition (FDR-corrected *P* value = 1.49 × 10^–05^) (Supplementary Fig. S2A). The IPA analysis showed the involvement of the following canonical pathways, G1/S checkpoint regulation (FDR-corrected *P* value = 1.37 × 10^–10^), PI3K/AKT signalling (FDR-corrected *P* value = 2.48 × 10^–10^), ovarian cancer signalling (FDR-corrected *P* value = 3.37 × 10^–10^), and PTEN signalling (FDR-corrected *P* value = 6.87 × 10^–10^), with molecular mechanisms of cancer (FDR-corrected *P* value = 2.09 × 10^–11^) at the top of the list (Supplementary Figs. S2B and S3A). According to the KEGG and WikiPathways databases, the PI3K-Akt signalling pathway was the most significantly enriched pathway. Moreover, enrichment analysis showed that DE miRNAs target genes were also implicated in the EGF/EGFR signalling pathway, STAT3 signalling pathway, G1 to S cell cycle control, MAPK signalling pathway, and mTOR signalling pathway (Fig. 2B and Supplementary Fig. S3B).

**Figure 3:**
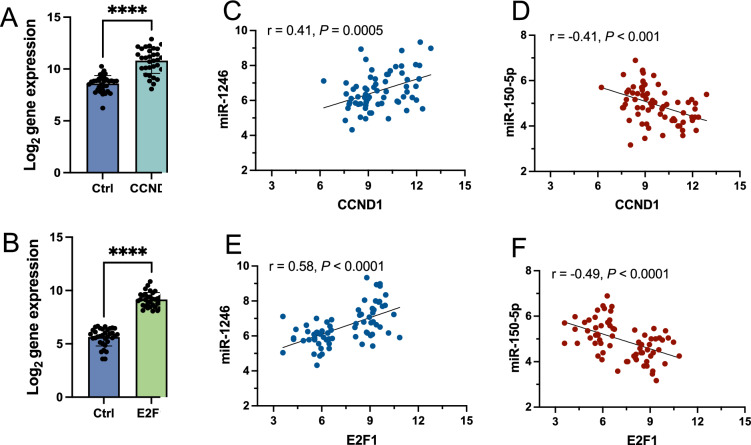
Expression level obtained by RNA-seq of (**A**) CCND1 and (**B**) E2F1 genes. All gene expression levels between HGSOC and non-cancer individuals were significantly different (****p < 0.0001). Pearson correlation of CCND1 expression with (**C**) miR-1246 expression and (**D**) miR-150-5p expression. Pearson correlation of E2F1 expression with (**E**) miR-1246 and (**F**) miR-150-5p expression.

Next, juxtaposed DE miRNAs target genes identified in our cohort with EOC-related from publicly available DisGeNET v7.0 database^41^. The 86 targets that overlapped between 1024 DE miRNA target genes and 1224 EOC-related genes were analysed in terms of known interactions using the HumanNet v3 platform^42^. Network analysis (Fig. 2C) was performed based on HumanNet-FN (functional gene network) which includes co-functional links (given by co-expression, co-essentiality, pathway database, protein domain profile associations, gene neighbourhood, and phylogenetic profile association) and protein–protein interactions. The top guide gene within this network was *E2F1* (score = 35.0), and the other guide genes within the top scores included: *CCND1*, *CDK4*, *CDK6*, *CCNE1*, *PTEN*, *ESR1*, *CCNE2*, *MYB*, and *CCND2*. *E2F1* encodes the E2F transcription factor 1, which regulates the expression of genes involved in cellular proliferation, differentiation, angiogenesis, DNA damage response, and apoptosis. Both genes, CCND1 and E2F1, were upregulated in HGSOC tissue (Fig. 3A and B). Furthermore, the correlation between the expression of the selected miR-1246 and miR-150-5p (with the lowest FDR value) in serum, and that of CCND1 and E2F1 was analysed in tumour tissue. The results, as presented in Fig. 3C–F, demonstrate that the expressions of miR-1246 and CCND1, as well as miR-1246 and E2F1, are moderately positively correlated (r = 0.41 and r = 0.58, respectively) with a significant statistical value of (p < 0.01). Conversely, a moderate negative correlation was observed between miR-150-5p and CCND1, and miR-150-5p and E2F1. The correlations were also statistically significant in these instances (p < 0.01). Within the obtained network, we also identified downstream candidate genes that could be functionally connected to the 86 input guide genes. The top scores comprised *CDKN2A*, RELA, *CDK1*, *RB1*, *CDKN2A*, *EP300*, *CDKN1B*, *PRKACA* and *CREBBP* with *CDK2* and *TP53* as top candidates (score = 49.6 and score = 44.5, respectively).

**Figure 4:**
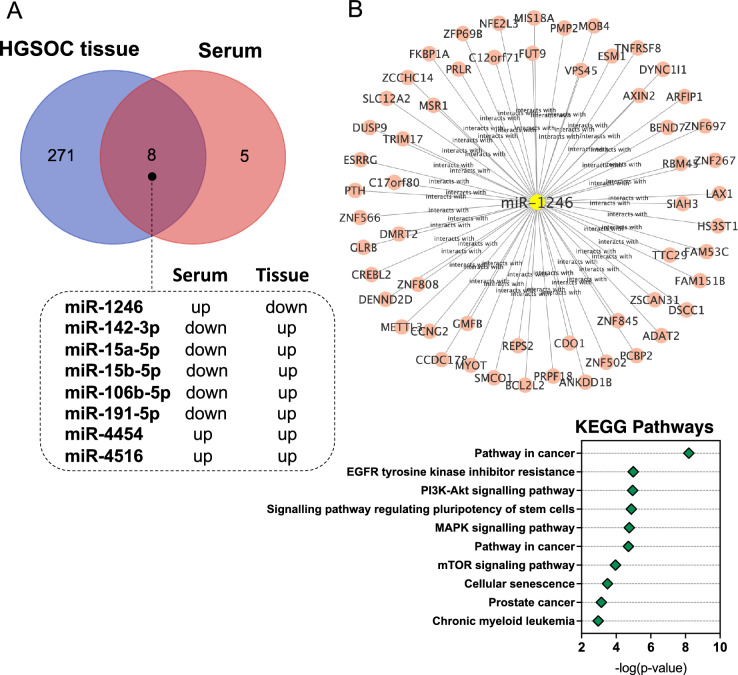
Comparison of miRNA expression between matched serum and tissue HGSOC samples. (**A**) Venn diagram of the list of DE miRNAs between serum and tissue samples. The diagram shows that eight miRNAs were significantly differentially expressed compared to healthy controls in serum and tissue samples. In addition, six out of eight miRNAs show an opposite trend of regulation, and two show a concordant trend; (**B**) Cytoscape network of miR-1246 and its targets and KEGG pathway analysis of miR-1246 target genes. All functional annotations were performed with the hypergeometric test and Bonferroni adjustment (corrected P value (FDR) ≤ 0.05); *KEGG* Kyoto encyclopedia of genes and genomes.

Finally, we examined the miRNA profile in ovarian cancer tissues matching previously screened serum samples. We performed a small RNA-seq analysis in matched serum and tumour tissue specimens. Analysis revealed 278 differentially expressed miRNAs (DEMs) (132 upregulated and 146 downregulated) in tissue compared to 13 found in serum. As shown in Fig. 4A, only eight DE miRNAs appeared to be differentially expressed in matched tissue and serum samples. Of these, the expression of miR-1246 was significantly upregulated in serum from cancer patients, whereas it was downregulated in cancer tissue compared to the control. In addition, we found a significant over-representation of miR-1246 targets in several KEGG pathways including pathways in cancer (FDR-corrected *P* value = 6.37 × 10^–09^), EGFR tyrosine kinase inhibitor resistance (FDR-corrected *P* value = 2.02 × 10^–06^), PI3K-Akt signalling pathway (FDR-corrected *P* value = 1.06 × 10^–06^), signalling pathway regulating pluripotency of stem cells (FDR-corrected *P* value = 6.04 × 10^–05^), and MAPK signalling pathway (FDR-corrected *P* value = 1.76 × 10^–05^) (Fig. 4B).

### Construction of a Weighted Gene Co-Expression Network and correlation with clinical traits

Using the Weighted Gene Co-Expression Network (WGCNA) algorithm^43^, we identified key miRNA modules in HGSOC. We used the normalised NanoString data to incorporate 798 miRNAs detected in 34 patients with HGSOC and 36 normal controls for the analysis (Supplementary Fig. S4A). After a series of quality assessments for the miRNA’s matrix, we set the soft threshold as 5 to construct and validate the scale-free network (Supplementary Fig. S4B). Five modules were identified by setting the cut height as 0.25 to merge similar modules (Supplementary Fig. S4C). Next, the correlation between modules and clinical traits was investigated (Supplementary Fig. S5A). We identified the yellow module consisting of 26 miRNAs that were highly correlated with HGSOC, FIGO stage, tumour size, nodules and metastasis (TNM). Of the 26 miRNAs present in the yellow module, eight were previously identified as DE miRNAs in Fig. 1. Next, we analysed 188 miRNA target genes from the yellow module. The top pathway identified by KEGG and WikiPathway analyses was the PI3K-Akt signalling pathway (Supplementary Fig. S5C). Among the affected processes, we also found the EGF/EGFR signalling pathway, mTOR signalling pathway, FoxO signalling pathway, and MAPK pathway.

### Evaluating circulating miRNAs as diagnostic markers for HGSOC

First, we performed receiver operating characteristic (ROC) analyses to assess the diagnostic performances of the 13 DE miRNAs to detect HGSOC. Corresponding area under the curve (AUC) values, confidence intervals (CI), sensitivity and specificity for cut-off points were calculated and are shown in Supplementary Table S2 and Supplementary Fig. S6. Four miRNAs, miR-1246, miR-150-5p, and miR-4454 + miR-7975 showed the highest AUCs reaching 0.923, 0.872, and 0.856, respectively. The occurrence, sensitivity and specificity for all DE miRNAs above 0.75 indicate that they possess good diagnostic potential.

### Identification of the best combination of miRNAs for HGSOC detection

To reveal the biological function of the DE miRNAs classification power for discrimination between HGSOC and non-cancer samples, we randomly divided 70 samples into two groups, the training set (70%) and the testing set (30%) (Table 1). Based on the changed expression of miRNAs in cancer and healthy patients identified by the NanoString method, we combined two attribute selection methods, Information Gain and Correlation-based Feature Subset Selection (CfsSubsetEval), to select the strongest miRNAs candidates to develop a diagnostic classification model. Both methods were carried out in the testing set with the use of leave-one-out cross-validation (LOOVC). The Information Gain method with the Ranker Search method is based on the calculation of decreasing entropy by adding attributes and Correlation-based feature selection prioritises uncorrelated features. The top 3 attributes by InfoGainAttributeEval Algorithm, best-explaining data, are miR-1246, miR-144-3p, and miR-150-5p. The top attributes by CfsSubsetEval Algorithm, are miR-1246, miR-150-5p, miR-144-3p, miR-4454 + miR-7075, and miR-4516. The strongest attributes, which were selected as overlapping from two attribute selections, were miR-1246, miR-150-5p, and miR-144-3p. All results are listed in Supplementary Table S3.

**Figure 5:**
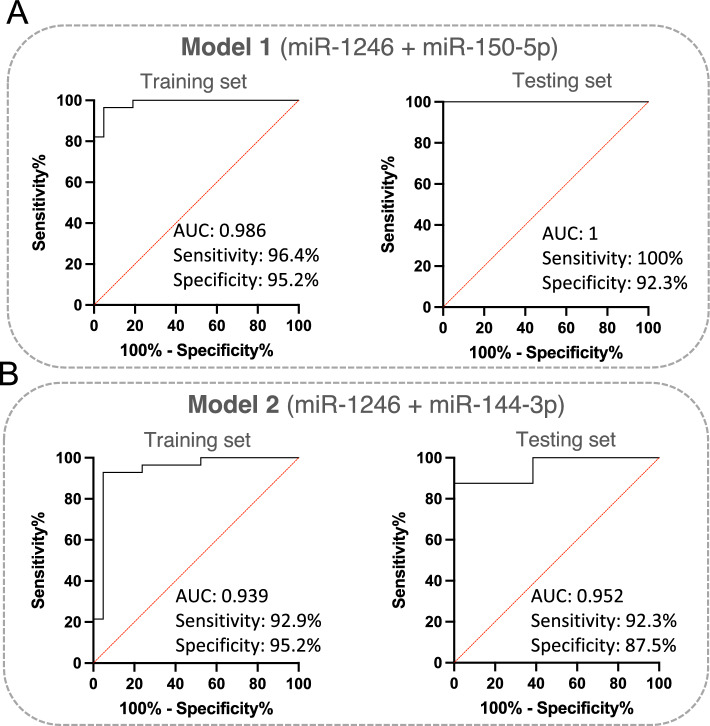
Development of the HGSOC diagnostic model. (**A**) ROC curve and AUC (area under the curve) for the training and the testing set for the diagnostic classification model based on data on the miR-1246 and miR-150-5p expression levels obtained using the NanoString platform. The graph consists of the AUC value, sensitivity and specificity corresponding to that point; (**B**) ROC curve and AUC (area under the curve) for the training and the testing set for the diagnostic classification model based on data on the miR-1246 and miR-144-3p expression levels obtained using the NanoString platform. The graph consists of the AUC value, sensitivity and specificity corresponding to that point.

To assess the diagnostic values of the selected above circulating miR-1246, miR-150-5p, and miR-144-3p, the multivariate logistic regression method was applied to develop the diagnostic models of the miRNAs under combination conditions. To develop stable models in the course of training, k-fold cross-validation (K = 10) was used. Based on the normalized NanoString data, two models were derived. Model 1 built on the expression of miR-1246 and miR-150-5p as independent variables, while Model 2 included miR-1246 and miR-144-3p (Supplementary Table S4). The ROC curves, and the AUCs values (Supplementary Table S5), as well as confusion matrices (Supplementary Table S6), were used to evaluate the diagnostic potential of both combination miRNAs in the training set and the testing set. The AUC was 1, the diagnostic sensitivity was 100% and the specificity was 92.3% for Model 1 (Fig. 5A), and the AUC was 0.938, with a sensitivity of 92.3% and specificity of 87.5% for Model 2 in the testing set (Fig. 5B). These results indicated that Model 1 including the combination of miR-1246 and miR-150-5p showed higher sensitivity and better specificity than Model 2 and can be more effective in the diagnosis of HGSOC.

### Validation of the diagnostic power of miR-1246 and miR-150-5p by RT-qPCR

**Figure 6:**
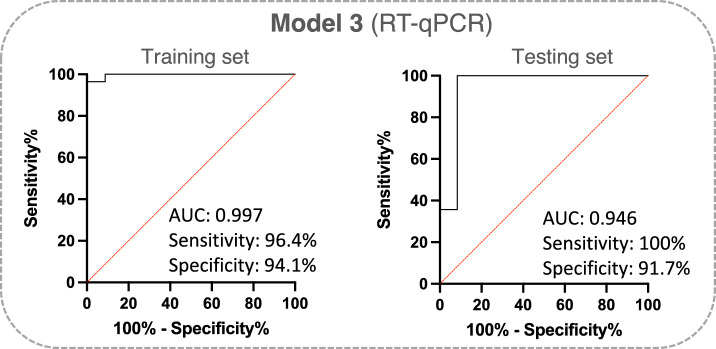
Validation of diagnostic power of miR-1246 and miR-150-5p by RT-qPCR method. ROC curves and AUC for the training and the testing set for the diagnostic classification model were obtained based on data obtained using the RT-qPCR technique. The graphs contain the AUC value, sensitivity and specificity corresponding to that point.

Next, to validate the diagnostic values of circulating miR-1246 and miR-150-5p from Model 1, we performed RT-qPCR analysis. For this, we used an enriched group of serum samples from 42 patients with HGSOC and 46 healthy donors (Table 1). The expression levels of miR-1246 and miR-150-5p were normalized to the expression of reference miRNAs, miR-103-3p and miR-199-5p. Based on the normalized RT-qPCR data, a diagnostic classification Model 3 was developed, which was trained on a training set comprising 70% of the data and validated on a testing set (30% of the data). To develop a stable diagnostic classification model, LOOCV was used in the course of training. The results of the diagnostic performance are shown in Fig. 6 and in Supplementary Table S7, Supplementary Table S8 and Supplementary Table S9. Model 3 provided very good discriminatory power on the training set (AUC 0.997), with a sensitivity of 96.4% and specificity of 94.1% and it highly classified patients in the testing set (AUC 0.946) with a perfect sensitivity of 100% and specificity of 91.7%.

### Validation of the diagnostic power of miR-1246 and miR-150-5p in an external dataset

**Figure 7:**
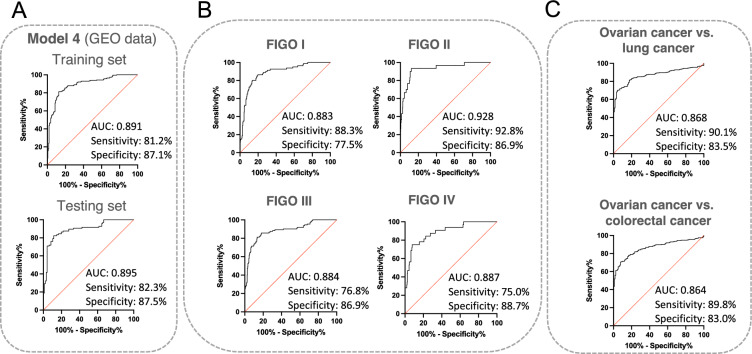
Validation of the HGSOC diagnostic model based on external data. (**A**) ROC curve and AUC for the training and the testing set for the diagnostic classification model based on public data on the miR-1246 and miR-150-5p expression levels obtained using an array technique, which is included in Gene Expression Omnibus (GEO) database. The graph consists of the AUC value, sensitivity and specificity corresponding to that point; (**B**) ROC curves and AUC for the diagnostic classification model depend on the ovarian cancer stage (FIGO I–IV). The graph contains the AUC value, sensitivity and specificity corresponding to that point; (**C**) ROC curves and AUC for the diagnostic classification model depend on the type of cancer. The graph consists of the AUC value, sensitivity and specificity corresponding to that point.

Next, additional validation of the optimised miR-1246 and miR-150-5p signature was performed using an external dataset (GSE 106,817, 3D-Gene Human miRNA V21_1.0.0 microarrays, Toray Industries.Inc.), which contains miRNA profiles of 4046 cancer patients and 2759 healthy donors. From the available set, the data of patients with ovarian cancer (n = 320) and the data of matched healthy donors (n = 320) were selected. The whole cohort was divided into a training set (70%) and a testing set (30%). We found that Model 4, created based on miR-1246 and miR-150-5p expression data in the GEO dataset, sufficiently discriminated between OC patients and healthy donors with AUC 0.891, sensitivity 81.2%, and specificity 87.1% in the training set and AUC 0.895, sensitivity 82.3% and specificity 87.5% in the testing set (Fig. 7A, Supplementary Table S10, Table 3 and Table S11). In addition, based on the FIGO criteria, we also subdivided ovarian cancer data according to the stage, including patients with early stages of cancer (FIGO 1 and 2), which are generally difficult to detect during routine clinical screening. The results of the diagnostic performance are shown in Fig. 7B and Table 3. Based on these results, we were able to discriminate between HGSOC at all FIGO stages and healthy donors with AUC values higher than 0.88 and sensitivity in the range of 75.0–92.8% and specificity in the range of 77.5–88.7%. Given that the dataset lacks an adequate description of histopathological subtypes for each sample, however, the source publication^45^ includes histology distribution. Most of the samples are HGSOC (182 out of 320); the rest include clear cell, endometroid and other epithelial and non-epithelial carcinoma.

**Table 3:** Quality parameters for diagnostic classification model 4 in training and test sets for expression measurements by microarray data (GEO dataset).

Name	miR-1246, miR-150-5p	miR-1246, miR-150-5p
Set	Training	Testing
Area under curve (AUC)	89.1%	89.5%
Confidence interval (CI) lower limit	86.1%	84.8%
CI upper limit	92.1%	94.1%
Cut-off point (Youden index)	0.56	0.56
Sensitivity	81.2%	82.3%
Specificity	87.1%	87.5%
FIGO I (n = 80), AUC (95% CI)	–	88.3%
FIGO II (n = 30), AUC (95% CI)	–	92.8%
FIGO III (n = 112), AUC (95% CI)	–	88.4%
FIGO IV (n = 32), AUC (95% CI)	–	88.7%

Finally, to investigate whether the serum miR-1246 and miR-150-5p profiles can distinguish ovarian cancer from other solid cancers, we developed a model based on two miRNA expression datasets in lung and colon cancers from the GSE 106,817 dataset. As shown in Fig. 7C, the trained models were able to properly discriminate patients with ovarian cancer from patients suffering from lung cancer (AUC 0.868, sensitivity 90.1%, and specificity 83.5%) or colon cancer (AUC 0.864, sensitivity 89.8%, and specificity 83.0%).

## Discussion

Despite substantial improvement in the diagnosis of heterogeneous diseases such as EOC, effective screening methods for early detection are still urgently needed. To date, several mathematical models and diagnostic algorithms have been created using histopathology and protein-based biomarker analyses in serum. However, while multivariate classification tools such as ROMA, Ova1 and Overa® have been found to be helpful for the diagnosis of EOC in general, they lack high sensitivity and specificity as well as early diagnosis potential^27,44^. In this context circulating miRNA emerges as a particularly promising new EOC biomarker.

Here, based on miRNA expression profiles in the serum of HGSOC patients and healthy individuals, we propose a classification model that could improve the diagnosis of ovarian cancer. We found that a two-miRNA biomarker signature comprising miR-1246 and miR-150-5p can successfully differentiate between HGSOC patients and non-cancer individuals with the AUC 0.946 (95% CI 0.964–1.00).At the point of maximum classification accuracysensitivity was 100% with a specificity of 91.7% Our proposed diagnostic model utilizing serum miRNAs may be inexpensive, non-invasive and easily used clinically. To date, several studies have investigated using miRNAs as serum biomarkers for ovarian cancer, but there has been no combination of miR-1246 and miR-150-5p for diagnosing HGSOC. Moreover, analyses often include combined samples from various histopathological subtypes, such as serous, clear cell, endometroid, mucinous, and other epithelial carcinomas. For example, Yokoi et al.^45^ constructed a diagnostic model based on the expression levels of nine serum miRNAs (miR-1246, miR-663b, miR-4730, miR-642a-3p, miR-658, miR-486-3p, miR-1207-5p, miR-4419b, miR-6124) for discriminating ovarian cancer patients from healthy women (AUC, 0.86; S, 82%; Sp, 91%). Unfortunately, this model could not efficiently discriminate patients with benign or borderline tumours from OC patients. Kan et al.^46^ identified a multivariate model combining miR-200b and miR-200c with good predictive power to discriminate patients with serous ovarian cancer, including both high- and low-grade types and healthy controls (AUC, 0.784). Similarly, multivariate analysis by Song et al.^47^ revealed the diagnostic potential of miR-26b and miR-21 with an AUC of 0.916, a sensitivity of 87.5% and a specificity of 90.4%. Halvorsen et al.^48^ proposed miR-200a-3p and miR-200c-3p as EOC detection biomarkers (AUC, 0.89; Sn, 84%, Sp, 83%). In another study by Todeshini et al.^49^, over 230 serum samples from OC and healthy controls were screened by microarrays for miRNA marker identification. According to the results, miR-1246, miR-595, and miR-2278 were significantly over-expressed in the serum of HGSOC patients compared to healthy controls. Interestingly, ROC curve analysis revealed miR-1246 as the best predictor, with an AUC of 0.82, a sensitivity of 87%, and a specificity of 77%. In this case, the combination of three biomarkers did not increase the AUC. In our study, for the first time, we showed that a model based on combining the expression of miR-1246 and miR-150-5p indicates promising outcomes in discriminating HGSOC patients from normal controls with higher predictive values. We have also evaluated the diagnostic power of these two miRNAs in an external dataset published by Yokoi et al.^45^. The evaluation model evaluation metrics (AUC, Sensitivity and Specificity) have decreased in this validation set, which might be due to the mixing of the histological OC subtypes in the dataset instead of only the HGSOC subtype, as in the discovery set. However, the resulting model is doing very well in setting the right OC diagnosis (AUC 0.895, sensitivity 82.3% and specificity 87.5%). Therefore, although the model based on miR-1246 and miR-150-5p was adjusted for HGSOC diagnosis, there is also potential in using it to recognise other subtypes of OC, however less effectively. Additionally, our data revealed that ovarian cancer patients could be sufficiently discriminated from those with lung and colorectal carcinoma. This information about analyses across various cancer types will also be helpful in the clinical application of serum miRNA panels for monitoring HGSOC.

Based on our results, we also noticed that miR-1246 was downregulated in tumour tissue samples, with a simultaneous upregulation in serum HGSOC patients. Circulating miRNA levels may reflect the condition of tumour tissue^50^. Thus, it can be considered that miR-1246, as a tumour suppressor, is released from tumour cells. Increasing evidence has emerged that miRNAs are locally produced in solid tumours and secreted into several body fluids, including serum^51,52^. Based on this evidence, it is believed that such miRNAs might be significant biomarkers for several types of cancer with high success and repeatability^53–55^. Serum levels of miR-1246 have been found to be the most upregulated miRNA in the serum of patients with lung cancer^56^. Similarly, the following studies in clinical settings have shown upregulation of miR-1246 in the serum or plasma of patients with colorectal cancer (CRC), prostate cancer, breast cancer, hepatocellular carcinoma (HCC), esophageal cancer, laryngeal squamous cell carcinoma (LSCC), and pancreatic cancer (PC)^57–65^. The diagnostic value of miR-1246 has been validated in different neoplastic disorders. The AUC values for various cancers ranged between 0.69 and 0.97. A very promising result can be seen in hepatocellular carcinoma and breast cancer, where miR-1246 has been used as a diagnostic marker with AUC values of 0.97 and 0.967, respectively^66,67^. The expression level of miR-150-5p in plasma samples has been shown to distinguish prostate cancer patients from healthy individuals with an AUC value of 0.817^68^. Likewise, Zou et al.^69^ reported the diagnostic potential of miR-150-5p in colorectal cancer with an AUC of 0.87.

In addition to miR-1246 and miR150-5p, we also identified several circulating DE miRNAs (miR-144-3p, miR-142-3p, miR-15a-5p, miR-15b-5p, miR-126-3p, miR-4454, miR-7975, miR-191-5p, miR-4516, miR-630, and miR-106b-5p), which have been previously reported to play a crucial role in HGSOC and other types of cancers. Decreased expression of miR-150-5p, miR-106b-5p, miR-126-3p, miR-142-3p, and miR-191-5p has been observed in presurgical plasma from serous epithelial ovarian cancer^62^. Importantly, miR-150-5p together with miR-106b-5p and miR-126-3p, have shown differential expression in benign presurgical plasma. Similarly, Todeschini et al.^49^ showed upregulated expression of miR-1246 along with downregulation of miR-106b-5p in miRNA profiling of serum samples from 168 HGSOC patients. In addition, they also reported significantly altered expression of miR-1246, miR-150-5p, miR-144-3p, miR-15a-5p, miR-15b-5p, miR-126-3p, and miR-106b-5p in matching HGSOC tissue. Previous research has also approved that serum levels of miR-142-3p were significantly lower in colorectal cancer (CRC) patients than in healthy controls. Furthermore, in this study, survival analysis demonstrated that CRC patients with low serum miR-142-3p levels had a lower 5-year overall survival rate^70^. Similar, to HGSOC, miR-150-5p was significantly downregulated in plasma samples from prostate cancer (PC) patients compared to cancer-free controls. A similar study by Carvalho et al.^71^ showed a lower expression level of miR-150-5p along with miR-142-3p in exosomes from breast cancer patients, which correlated well with the stage of the tumour.

Our functional annotation analyses of predicted DE miRNA targets revealed gene enrichment in key cancer pathways relevant to tumorigenesis including, PI3K/AKT signalling pathway, EGFR signalling pathway, MAPK pathway, STAT3 signalling pathway, PTEN signalling pathway, and regulation of the cell cycle transition from G1 to S phase. EGFR signalling promotes ovarian tumorigenesis including proliferation, migration and angiogenesis, and high EGFR expression correlates with poor prognosis^72,73^. A major downstream signalling pathway of EGFR is the phosphoinositide 3-kinase (PI3K)/protein kinase B (AKT) pathway, which plays a crucial role in OC tumorigenesis and is related to aggressive phenotypes, chemo- and radiotherapy resistance, and poor prognosis in OC^74–76^. Furthermore, the relationship between hyperactive EGFR signalling through STAT3 and PI3K activity together promotes high-grade ovarian cancer progression to cisplatin resistance^77,78^. By network-based analysis, we identified the top 10 hub genes, including *CCND1*, *E2F1*, *PTEN*, *STAT3*, *CDK4*, *CDK6*, and *GRB2*. Their expression might be regulated by various mechanisms, along with regulation by miRNAs, for instance, by miR-106a-5p, miR-150-5p, miR-15-5p, miR-144-30, miR-4516 and miR-1246^79,80^. Cyclin D1 (CCND1) is one of the cell cycle regulators modulating the development of malignant tumours. It has been reported that the *CCND1* gene can bind to cyclin-dependent kinase 4 (CDK4) and CDK6 to form a complex, which cooperates with the cyclin E-CDK2 complex and releases the transcription factor E2F, thus leading to an abnormal cell cycle and tumorigenesis^81^. PTEN is a major downstream effector of the PI3K pathway, a phosphatase capable of antagonising the PI3K/AKT pathway through the dephosphorylation of the phosphatidylinositol (3,4,5) – triphosphate (PIP3)^82^. PTEN also directly dephosphorylates AKT1, leading to the inhibition of tumorigenesis by regulating the PTEN/PIP3/PDK1-Akt signalling pathway. Leveraging the HumanNet-FN platform, we also identified top guide and candidate genes within the created networks based on overlapping putative target mRNAs identified for the serum miRNA profile and genes related to EOC by the DisGeNET database. We identified *E2F1,* which encoded E2F Transcription Factor 1, as the top guide gene. *E2F1* is well-known as a good prognostic indicator in ovarian carcinoma and its overexpression has been associated with unfavourable disease-free and overall survival^83,84^. Recent studies have shown that the *E2F1* gene can be regulated by the PI3K/AKT pathway. AKT has been shown to directly phosphorylate E2F1, which enhances its transcriptional activity and promotes cell proliferation^85^. In addition, the PI3KT/AKT pathway can regulate the expression of other genes that control the cell cycle, such as cyclin D1 and p27^Kip1^, which can, in turn, regulate E2F1 activity^86^.

Our study has several limitations. First, the sample size of this study was limited. Therefore, we further performed large-scale validation to determine if the constructed model was efficacious. Second, our model was built to predict diagnosis and cannot be used to predict prognosis or recurrence. Thus, a longitudinal study may clarify the effectiveness of our model for treatment effects and timely diagnosis. In addition, because over 80% of cases were stratified into FIGO III-IV stages, the proposed model will not be sufficient for early diagnosis, however, based on the results from the external dataset, there is potential, that the model is as discriminative for high as for low FIGO stage. In summary, a large-scale study is now required to confirm the role of these miRNAs as biomarkers for HGSOC.

Given that OC is a heterogeneous disease frequently lacking symptoms at the early stage and with later metastasis stages often misinterpreted as related to digestive system malfunctions, improved diagnostic tools are needed. Tissue biopsy is the gold standard for the diagnosis of a variety of tumours. However, in the case of OC, tissue biopsies have to be avoided because puncture can cause cancer cells to disseminate into the peritoneal cavity, promoting peritoneal metastasis. We report 12 DE serum-derived miRNAs in HGSOC patients and their functional analysis. In this study, we have also developed a diagnostic model based on the expression of two serum-circulating miRNAs, which shows high sensitivity and specificity for identifying HGSOC patients. The developed diagnostic model might be an important tool in the future of gynaecologic oncology as non-invasive diagnosis support.

## Methods

### Study cohort

Serum samples were collected in the Clinical Hospital in Bialystok by Biobank at the Medical University of Bialystok, with high standards of strict biobanking procedures described further by Niklinski et al.^87^, between 2015 and 2019. Finally, the present study involved 70 patients, including 34 patients with HGSOC and 35 non-cancer individuals for NanoString screening analysis, and 88 patients, including 42 patients with HGSOC and 46 healthy controls for RT-qPCR validation (Table 1). The serum was collected at baseline before surgery and any treatment along with the clinical information about patients’ age, BMI CA125 level, FIGO stage and TNM stage. Women diagnosed with types of cancer other than high-grade serous ovarian cancer, chemotherapy or radiotherapy before serum collection, and material containing at least 50% tumour cells were excluded from the study. According to the recommendation of the International Federation of Gynaecology and Obstetrics^88^, patients were stratified into FIGO I FIGO II, FIGO III, and FIGO IV stages in the NanoString and the RT-qPCR analysis. Samples from healthy donors with no cancer history were obtained from the Clinical Research Centre, Medical University of Bialystok. Serum was harvested from patients by centrifugation of blood in serological tubes and stored at − 80 °C until further use. Sample collection was approved by the local ethics committee of the Medical University of Bialystok, Poland (approval number: R-I-002/36/2014 and APK.002.69.2020) and conducted according to the Declaration of Helsinki. All participants provided written informed consent.

### Sample size estimation

Based on our previous experiments and pilot data, we calculated the minimal number of samples per experimental group (tumour or normal) to detect two-fold differences in relative expression levels between groups at true positive detection powers of 80% and 90%^89^. Since tumour and normal tissue differ in terms of inter-individual variations, we used the RNASeqPower R package to apply the statistics data covering obtained real counts and coefficients of variation per group. For the NanoString nCounter miRNA data, we estimated that to obtain 80% power, we would need nine samples per group, whereas to obtain a high power of 90%, we would need 12 samples. For the smallRNA-Seq data, we estimated that to obtain 80% power, we would need 20 samples per miRNA group, whereas to obtain a high power of 90%, we would need 27 samples per miRNA group. Finally, our groups for NanoString nCounter and smallRNA-seq analyses consisted of 34 (normal tissue) and 36 (tumour tissue) samples, thus allowing for more than 90% power in any of the comparisons performed.

### RNA preparation and miRNA profiling by NanoString

RNA isolation with miRNA fraction serum samples was performed using the miRNeasy Serum/Plasma Advanced Kit (Qiagen, Germany) according to the manufacturer’s instructions. The analysis was performed using the nCounter® Analysis System (NanoString Technologies, WA, USA) and the nCounter Human v3 miRNA Panel. Briefly, as input material, three ng of isolated miRNA was used. Next, unique DNA tags were ligated onto the 3′ end of each mature miRNA, followed by overnight hybridization (65 °C) to nCounter Reporter and Capture probes. After hybridization, samples were placed into the nCounter Prep Station for sample purification and target/probe complex immobilization on the cartridge. For each assay, a high-density scan (555 fields of view) was performed on the nCounter Digital Analyser to count individual fluorescent barcodes and quantify target miRNA molecules in each sample.

### miRNA profiling by Next Generation Sequencing

Total RNA with miRNA fraction was extracted from tissue samples using *mir*Vana™ Isolation Kit (ThermoFisher Scientific, MA, USA) according to the manufacturer’s instructions. RNA concentration, purity and integrity were assessed by Qubit (Invitrogen, CA, USA) and Tape Station (Agilent Technologies, CA, USA). Small RNA-Seq libraries were constructed from 1 µg of total RNA with an RNA integrity number (RIN) > 8 using the Illumina TruSeq Small RNA Preparation Kit (Illumina, CA, USA). Indexed libraries were pooled, clustered using cBot and sequenced on the HiSeq 4000 platform, generating 50 bp single-end reads (1 × 50 bp). Sequencing data were processed to obtain fastq files with the bcl2fastq pipeline (Illumina, San Diego, USA), including demultiplexing and adapter trimming steps. The quality of the obtained reads was assessed using FastQC (Babraham Institute, Cambridge, United Kingdom) and multiQC^90^ before the analysis, as well as after different processing steps. Reads were trimmed from adapter sequences with cutadapt^91^ and only reads that contained adapters were kept for further analysis. After adapter trimming reads were filtered using cutadapt for quality and length in the range of 16–28 bp. MiRNA detection was performed with miRge 2.0^92^ using bowtie for alignment and miRBase v22^93^. Raw miRNA counts were used as input for DE miRNA analysis. MiRNA count data were filtered for lowly expressed miRNAs of less than five raw counts in the smallest library, taking into account corresponding CPM values (Counts per Milion). Counts were normalized using a weighted trimmed mean of the log expression ratios (trimmed mean of M values – TMM)^94^. Data were further transformed for linear modelling using voom^95^. Linear modelling and empirical Bayes moderation were applied using the limma package to assess the differential expression of miRNAs^96^. P values were corrected for multiple comparisons with FDR. The results plots were created in the R environment and basic statistical functions.

### qPCR

miRNA extraction from serum samples was performed as described previously. Reference miRNAs with stable expression across all samples, hsa-miR-103-3p and hsa-miR-199b-5p, were selected based on the NanoString data using the NormFinder algorithm^97^. The 13 DE miRNAs and two reference miRNAs were profiled using the miRCURY LNA SYBR Green PCR Kit (Qiagen, Germany). The miRCURY LNA RT Kit (Qiagen, Germany) was used for the reverse transcription reaction. The reaction was conducted with the primers as shown in Table S12. The temperature profile of the qPCR reaction was as follows: 2 min at 95 °C and 50 cycles: 10 s at 95 °C and 60 s at 56 °C. Amplification was performed using the LightCycler 480 (Roche, Switzerland). Subsequently, PCR threshold cycles (C_t_) of the tested miRNAs and reference miRNAs were determined for the tested samples and the calibrator. The relative expression for each miRNA was calculated with PCR efficiency correction^98^. Efficiency (E) was calculated from the slopes of the calibration curve according to the equation: E = 10 [–1/slope]. Reactions with amplification efficiency below 1.6 were removed. The relative expression ratio of a target miRNA was computed based on its PCR efficiencies (E) and the Ct value difference (Δ) of unknown group samples (test) versus the control group (Δ Ct control-test). The relative calculation was based on the MEAN Ct of the experimental group.

### miRNA target gene prediction and pathway analysis

The identification of DE miRNA target genes was performed using Ingenuity Pathway Analysis Software^31^ (IPA, Qiagen Inc. https://qiagenbioinformatics.com/products/ingenuity-pathway analysis), mirDB database^32^ (https://mirdb.org), and TagetScanHuman 8.0 database^33^ (https://www.targetscan.org/vert_80/). Gene Ontology Biological Process and KEGG Pathway analyses were conducted with the ClusterProfile R package^99^. IPA was used to perform the core analysis to identify canonical pathways. Over-representation analyses were performed using hypergeometric tests under α = 0.05 (*P*-values corrected with FDR). To construct a PPI network, we used STRING (https://string-db.org/; v.11.5). Genes with a confidence score ≥ 0.4 were chosen to build a network model visualized by Cytoscape v.3.9.1. Nine topological algorithms in plug-in *cytoHubba*^36^, consisting of “MCC”, “MNC”, “Degree”, “Bottle Neck”, “EcCentricity”, “Closeness”, “Stress”, and “Radiality” were selected to identify the hub genes in PPI analysis. Analyses of functional interaction networks were based on the HumanNet v3 platform by applying the HumenNet-FN (functional gene network) mode (https://www.inetbio.org/humannet/)^42^.

### WGCNA

Normalized counts according to the nSolver protocol were used as the starting point of the analysis. Counts were transformed to adjust for heteroscedasticity with log2 and precision weights based on the mean–variance trend. Hierarchical clustering was performed based on the transformed data (Supplementary Fig. S4A). The discovery and analysis of miRNA co-expression modules in the patient’s serum based on NanoString nCounter data were performed with Weighted Gene Coexpression Network Analysis (WGCNA)^100^ by application of the WGCNA R libraries^101^. Outliers were removed based on standardised connectivity. Pearson correlation was used in the signed network construction with soft threshold selection (R^2^ > 0.8) (Supplementary Fig. S4B, C). Modules were merged based on a remarkably high eigengene similarity correlation threshold (0.95). Gene modules to trait relationships were evaluated with Spearman correlation.

### Diagnostic model development

The normalised NanoString counts and RT-qPCR data were used for model development. First, both datasets were split into a discovery set (70%) and a validation set (30%) (Table 1). The splits have been random and have been done, as all further calculations in R version 3.6.1^102,103^. The attributes were selected using Waikato Environment for Knowledge Analysis (WEKA) version 3.8.3. (c) 1999–2018 The University of Waikato, Hamilton, New Zealand. Two independent algorithms InfoGainAttributeEval and Correlation-based Feature Subset Selection (CfsSubsetEval), were used on the training set to select the best classifiers. Feature selection via information gain using InfoGainAttributeEval was based on the calculation of decreasing entropy by adding attributes and selection attributes that most strongly reduce entropy. The CfsSubsetEval method evaluated the worth of a subset of attributes by considering the individual predictive ability of each feature along with the degree of redundancy between them. Both methods were carried out on the testing set using the LOOCV^104,105^. A multivariate logistic regression model was built using a training set. Training of the model was performed using repeated (n = 3) k-fold-cross-validation (k = 10) in R version 3.6.1 (R Core Team (2013) R: A Language and Environment for Statistical Computing. R Foundation for Statistical Computing, Vienna. https://www.R-project.org)^103^. To validate the model, the ROC curve and the AUC were calculated by the pROC package^106^. The cut-off point for the ROC curve was calculated by the Youden index. The confusion matrix including information about TP (true positives), TN (true negatives), FP (false positives), and FN (false negatives) has been prepared. The evaluation of model classification has been based on the testing dataset. The choice of miRNAs was evaluated using the independent, external and publicly available dataset GSE1089 from the Gene Expression Omnibus (GEO) database (data from: https://www.ncbi.nlm.nih.gov/geo/query/acc.cgi?acc=GSE106817), which contains miRNA profiles of 4089 cancer patients and 2759 healthy donors. These external data were generated using the 3D-Gene Human miRNA V21_1.0.0 (Toray Industries, Inc.) microarray platform. Patients with ovarian cancer (n = 320) were filtered from the dataset. Furthermore, 320 patients were randomly selected from the control dataset to balance the groups. Most of the population used for the validation of our model consisted of high-grade serous ovarian cancer (HGSOC) (n = 182). Other samples include clear cell (n = 64), endometroid (n = 43), other epithelial carcinoma (n = 17), and non-epithelial carcinoma (n = 13). As the datasets include data from another platform, the new model has been trained according to the above workflow. The evaluation also followed the same steps as previously described. In addition, according to the FIGO criteria, the GEO dataset was subdivided based on stage, including patients with FIGO I (n = 80), FIGO II (n = 30), FIGO III (n = 112), and FIGO IV (n = 32). A logistic regression model was developed for data for each stage according to the above workflow. The GEO dataset also includes patients with other types of cancers. Based on these data, the model was developed and examined based on the AUC calculated for datasets combined from ovarian cancer patients and lung cancer patients or colorectal cancer.

### Statistical analyses

Statistical analyses were performed using GraphPad Prism 9 (v.9.3.1) software. The Wilcoxon rank sum test (equivalent to the Mann–Whitney *U* test) was used to investigate the differences in BMI and age between the HGSOC patients’ group and the healthy donors. Raw miRNA data were analysed using nSolver Software v. 4.0 (NanoString Technologies, WA, USA). For technical variations, code-set content normalisation was performed relative to the ligation controls. Ratios were calculated by specifying the healthy volunteers’ samples as a baseline. Unpaired Mann–Whitney tests were performed to identify differences between patients and healthy controls. Correction for multiple testing was performed with False Discovery Rate (FDR) according to Benjamin-Hochberg. The Pearson correlation coefficient (*r*) was used to estimate the correlation between the identified DE miRNAs and clinical parameters.

### Ethics approval and consent to participate

All procedures of human samples were conducted after the approval of the local ethics committee of the Medical University of Bialystok, Poland (approval number: R-I-002/36/2014 and APK.002.69.2020) and according to the Declaration of Helsinki. All patients provided written informed consent for their participation in the study and their identities have been anonymised.

### Supplementary Information

Supplementary Figure S1.

Supplementary Figure S2.

Supplementary Figure S3.

Supplementary Figure S4.

Supplementary Figure S5.

Supplementary Figure S6.

Supplementary Tables.

Supplementary Legends.

## Data Availability

Datasets generated by the NanoString platform are deposited on GEO: GSE235525. Other datasets used and/or analysed during the current study are available from the corresponding author on reasonable request.
