# Supplementary figures and images for: Identification of serum miR-1246 and miR-150-5p as novel diagnostic biomarkers for high-grade serous ovarian cancer
Source: Sci Rep. 2023 Nov 7;13:19287. doi: 10.1038/s41598-023-45317-7 (PMC10630404; doi:10.1038/s41598-023-45317-7)

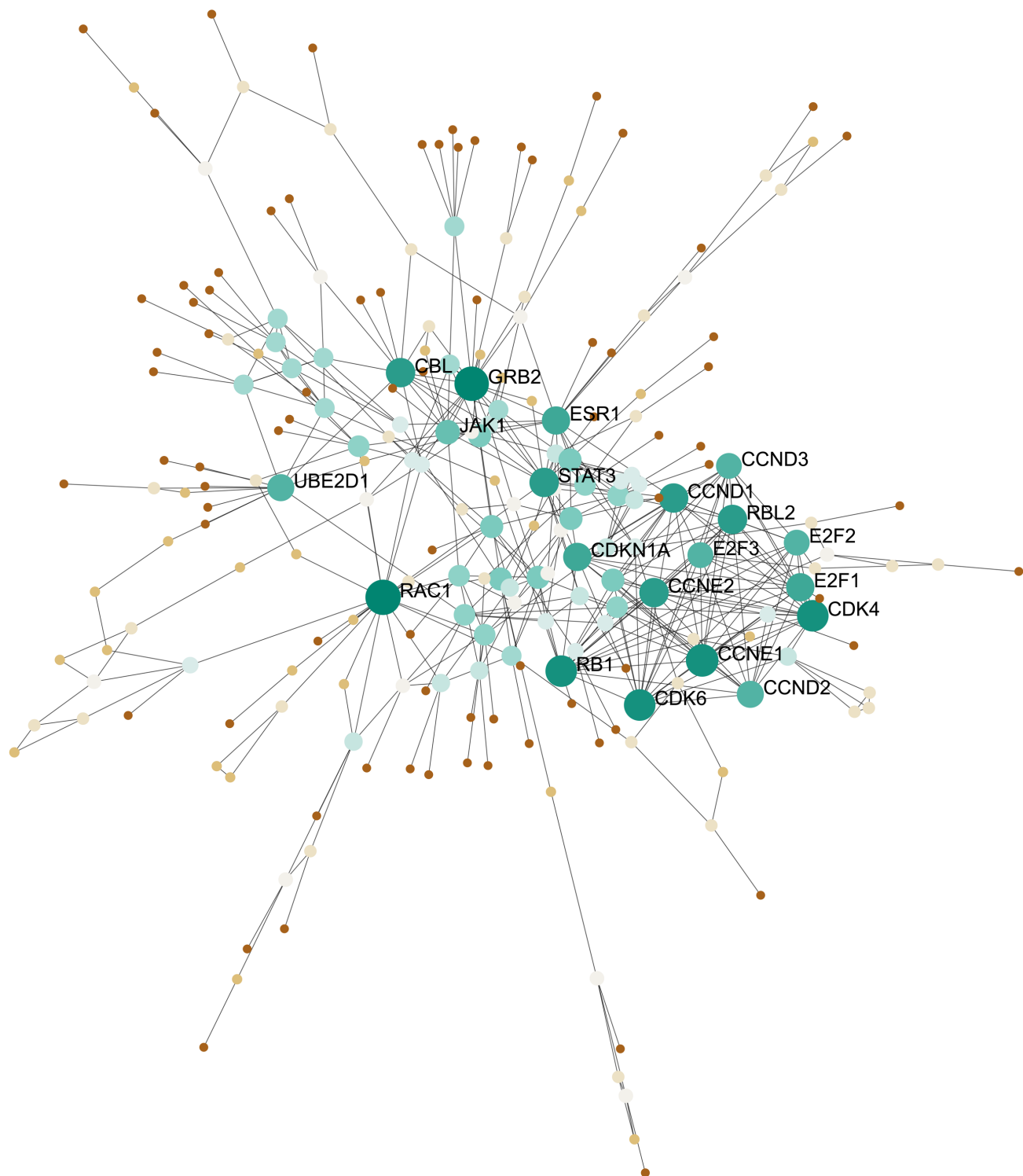

Supplement: Supplementary file 1 — Supplementary Figure S1. [file 41598_2023_45317_MOESM1_ESM.pdf]

A

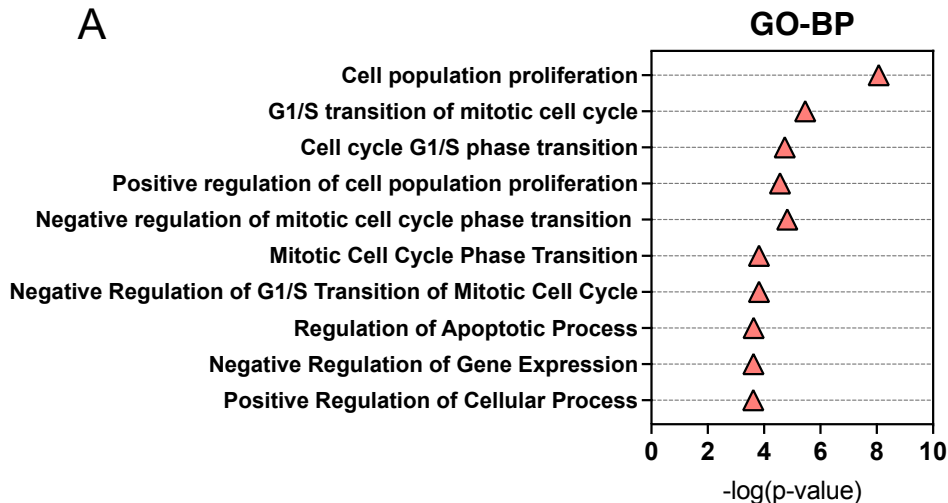

B

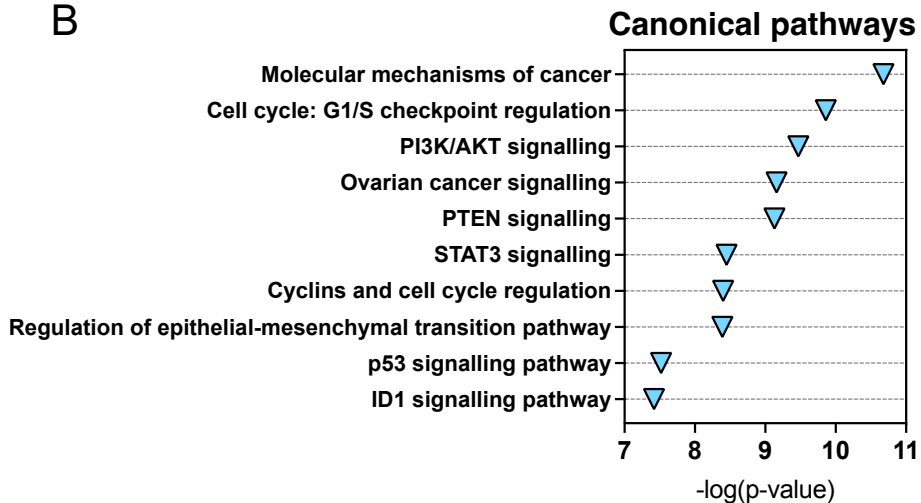

Supplement: Supplementary file 2 — Supplementary Figure S2. [file 41598_2023_45317_MOESM2_ESM.pdf]

A

Molecular Mechanisms of Cancer : Overlapped\_target

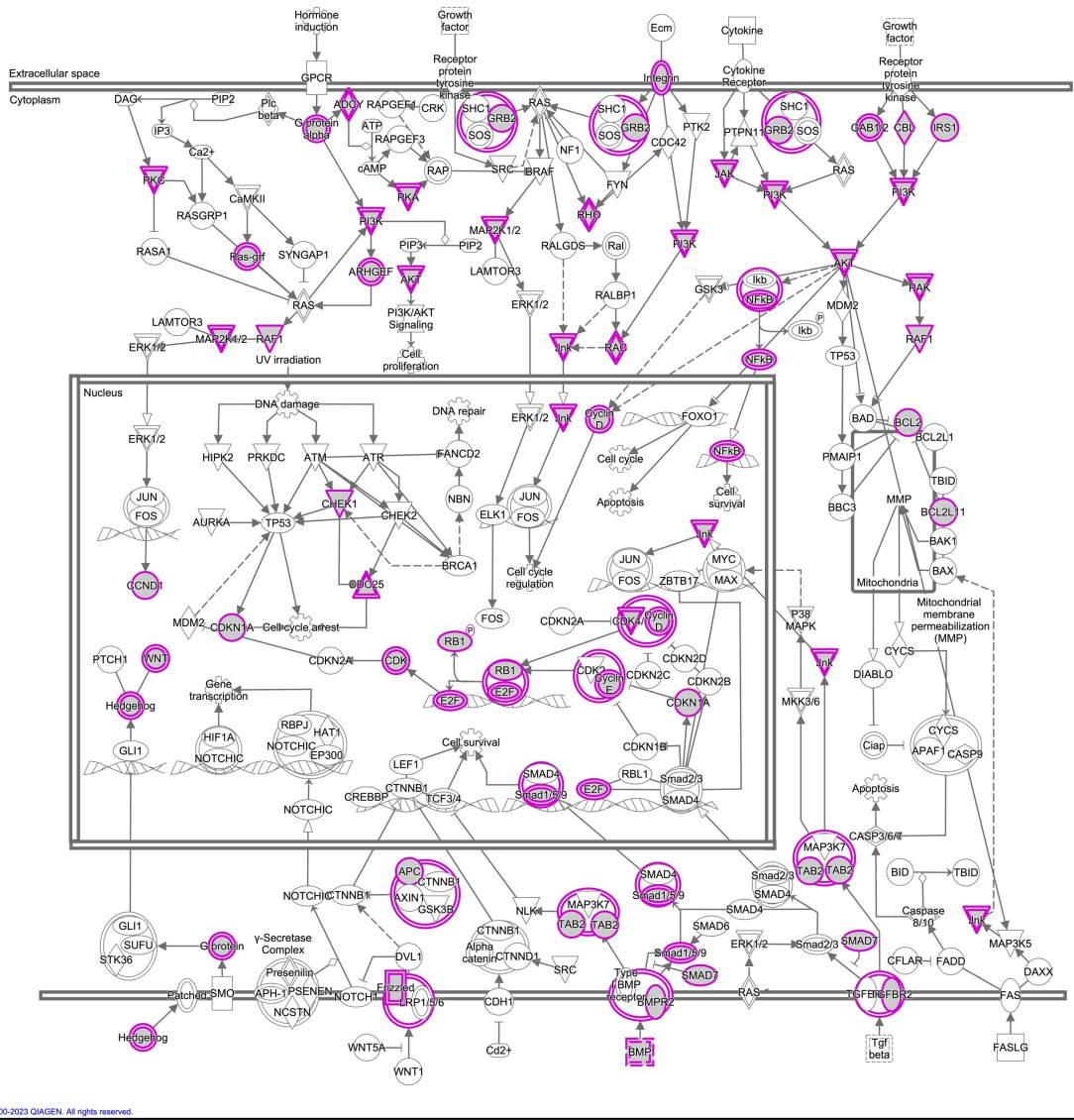

B

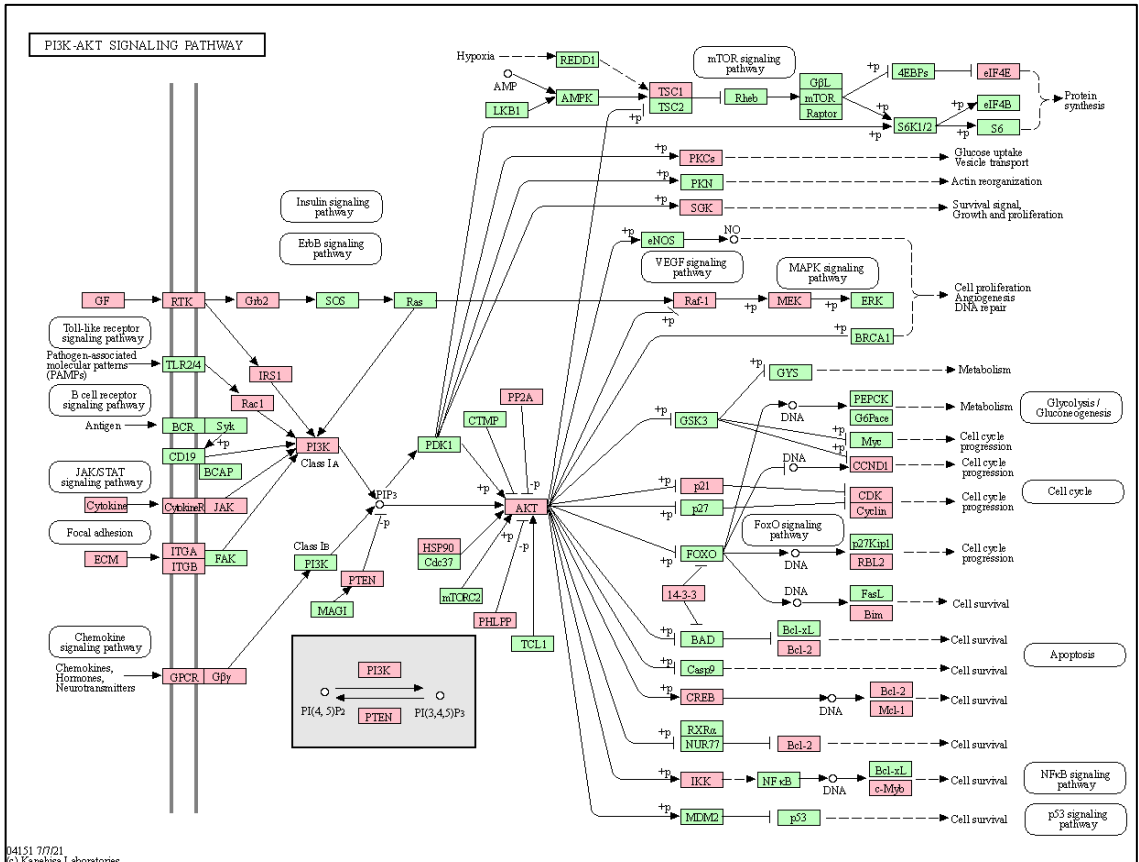

Supplement: Supplementary file 3 — Supplementary Figure S3. [file 41598_2023_45317_MOESM3_ESM.pdf]

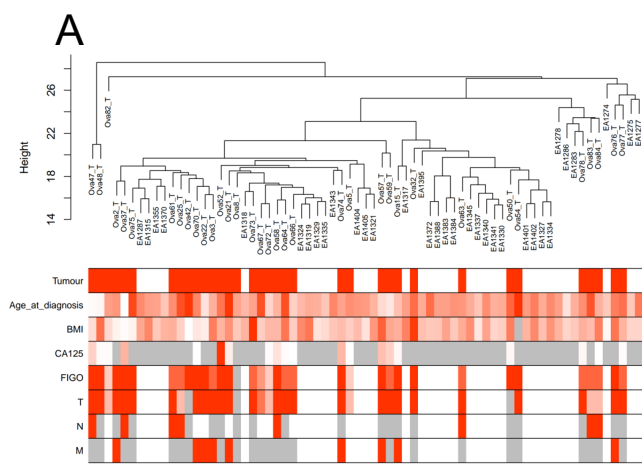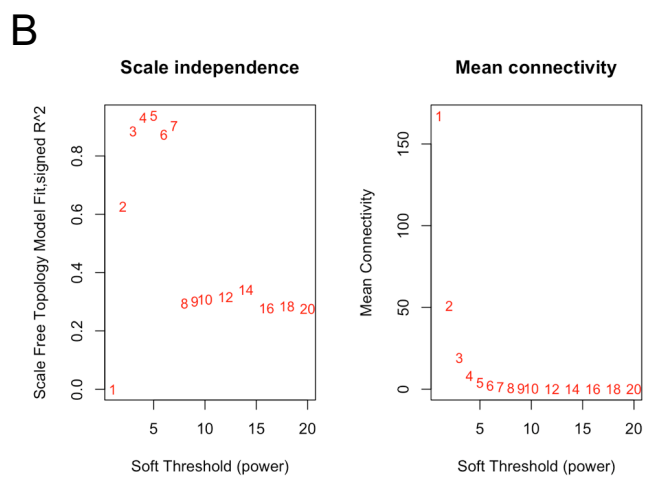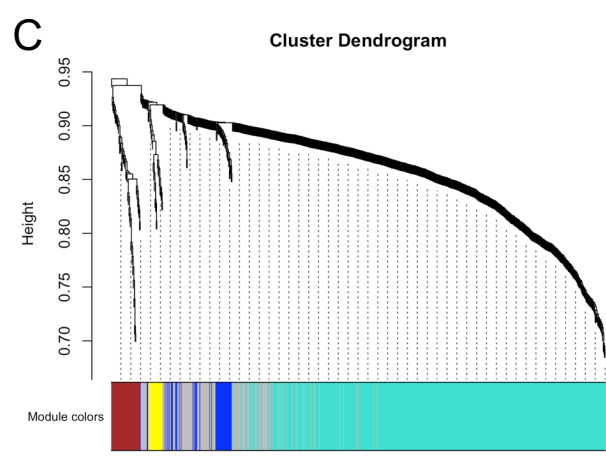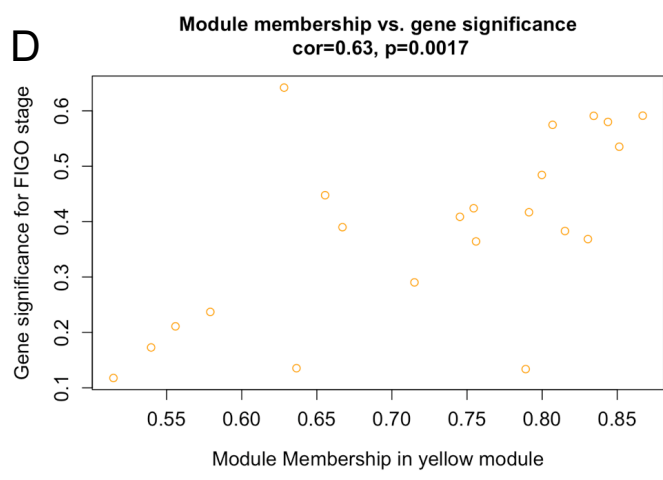

Supplement: Supplementary file 4 — Supplementary Figure S4. [file 41598_2023_45317_MOESM4_ESM.pdf]

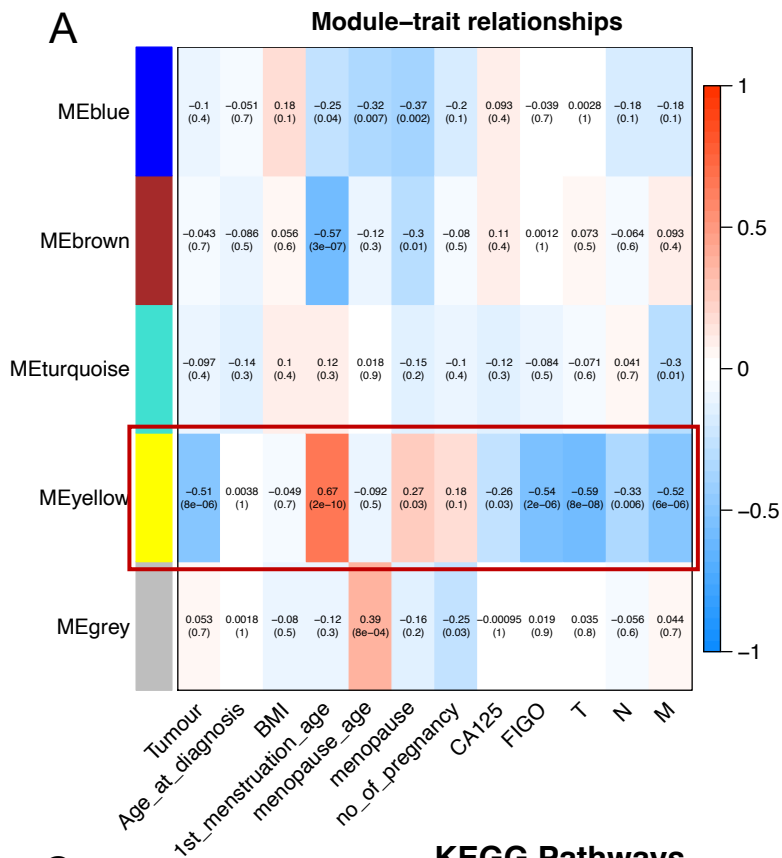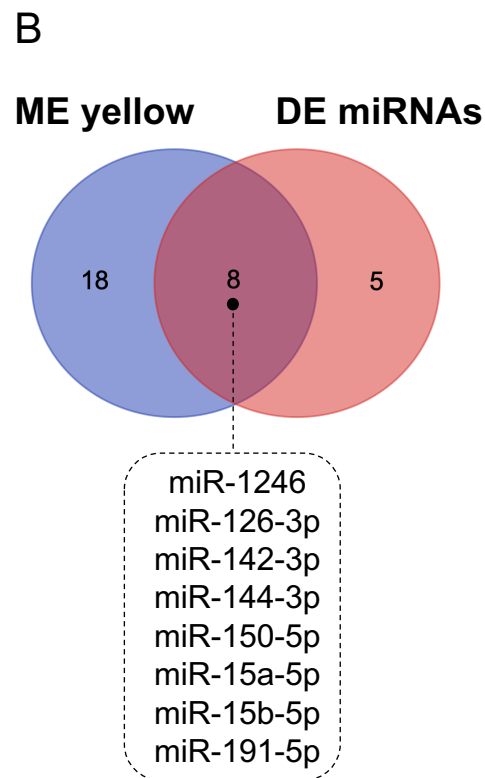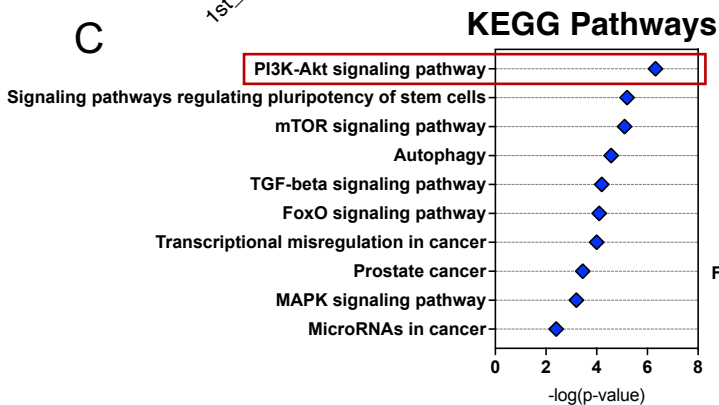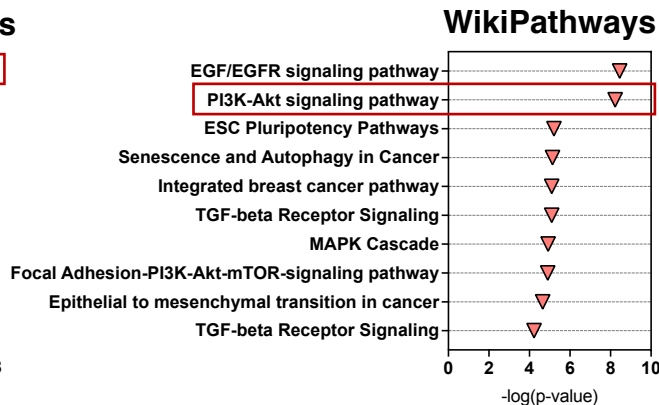

Supplement: Supplementary file 5 — Supplementary Figure S5. [file 41598_2023_45317_MOESM5_ESM.pdf]

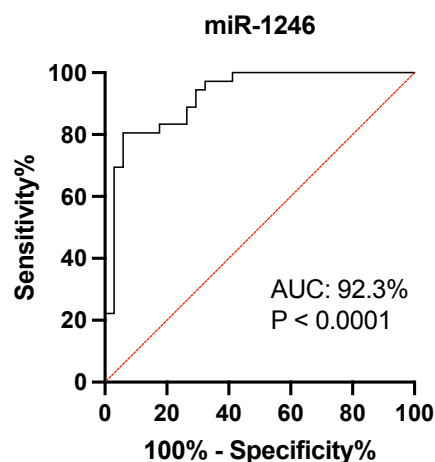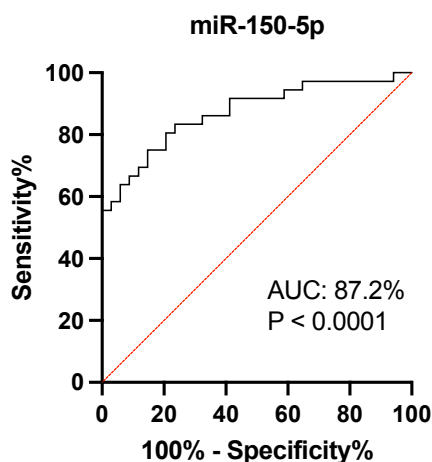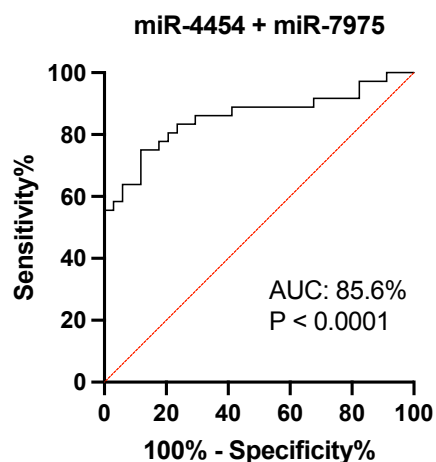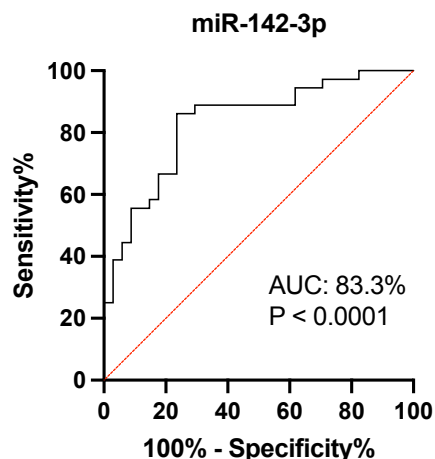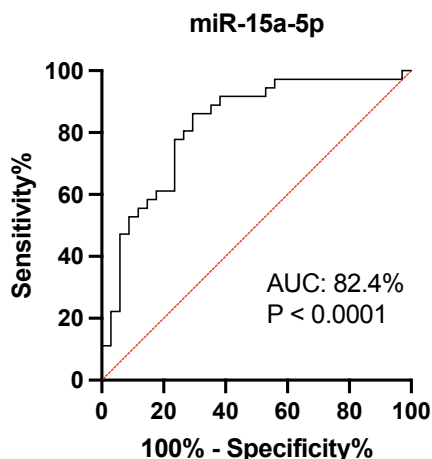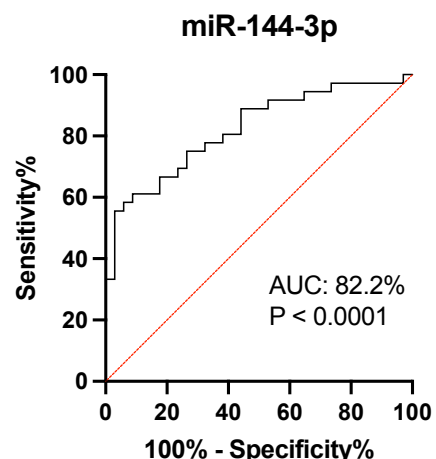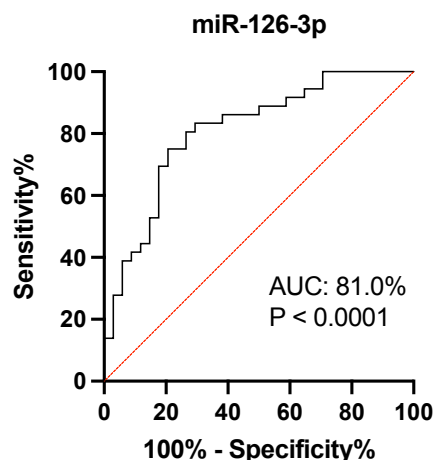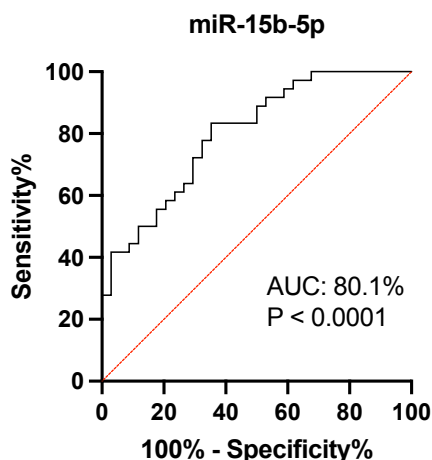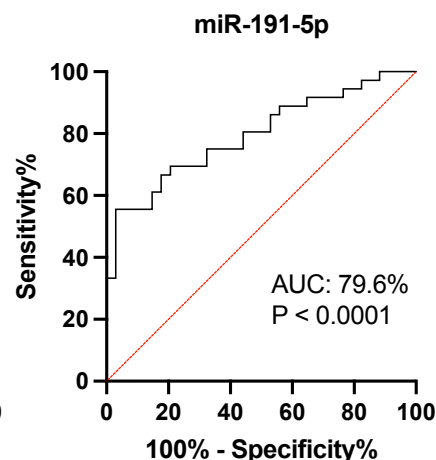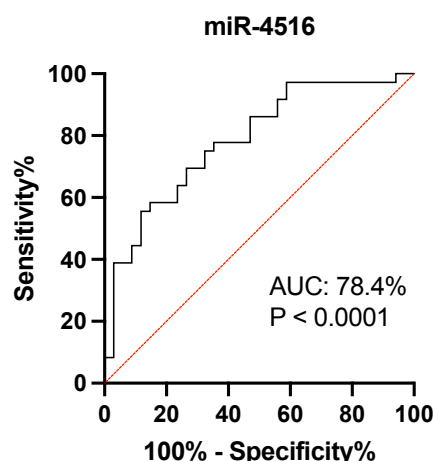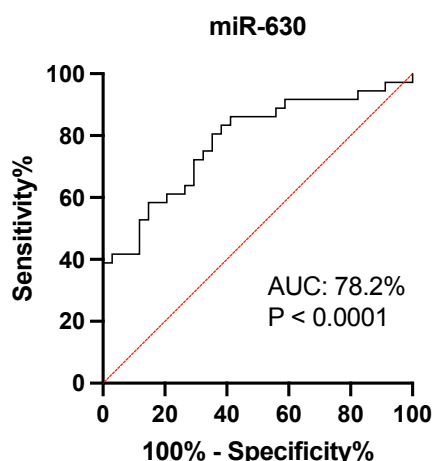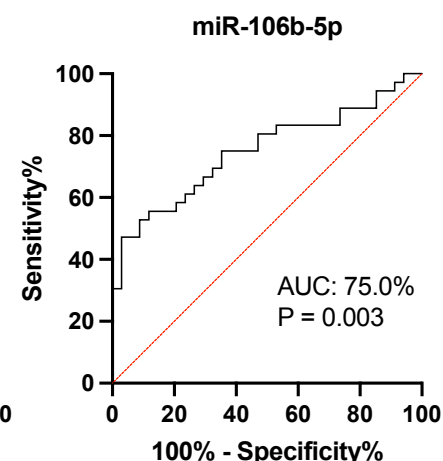

Supplement: Supplementary file 6 — Supplementary Figure S6. [file 41598_2023_45317_MOESM6_ESM.pdf]
