# Supplementary material for: Identification of serum miR-1246 and miR-150-5p as novel diagnostic biomarkers for high-grade serous ovarian cancer
Source: Sci Rep. 2023 Nov 7;13:19287. doi: 10.1038/s41598-023-45317-7 (PMC10630404; doi:10.1038/s41598-023-45317-7)
Supplement: Supplementary file 7 — Supplementary Tables. [file 41598_2023_45317_MOESM7_ESM.docx]

***Supplementary Information***

**Identification of serum miR-1246 and miR-150-5p as novel diagnostic biomarkers for high-grade serous ovarian cancer**

Magdalena Niemira^1*^, Anna Erol^1^, Agnieszka Bielska^1^, Anna Zeller^1^, Anna Skwarska^2^, Karolina Chwialkowska^3^, Mariusz Kuzmicki^4^, Jacek Szamatowicz^4^, Joanna Reszec^5^, Pawel Knapp^6^, Marcin Moniuszko^7^, Adam Kretowski^1^

*** Correspondence:** Corresponding Author: magdalena.niemira@umb.edu.pl

# SUPPLEMENTARY TABLES AND THEIR LEGENDS

**Supplementary Table S1.** Hub genes for miRNA targets ranked by different CytoHubba methods. MCC, the maximal clique centrality; MNC, the maximum neighbourhood component.

| **category** | **Rank methods in CytoHubba** | | | | | | | | | |
| --- | --- | --- | --- | --- | --- | --- | --- | --- | --- | --- |
|  | **MCC** | **MNC** | **Degree** | **BottleNeck** | **EcCentricity** | | **Closennes** | | **Stress** | **Radiality** |
| 1 | **CCND1** | **E2F1** | **CCND1** | **STAT3** | **CCND1** | **CCND1** | | **PTEN** | | **PTEN** |
| 2 | **E2F1** | **PTEN** | **E2F1** | **PTEN** | **PTEN** | **STAT3** | | **E2F1** | | **STAT3** |
| 3 | STAT3 | **CCND1** | **PTEN** | **E2F1** | **E2F1** | **E2F1** | | **STAT3** | | **CREB1** |
| 4 | **CDK4** | **STAT3** | **STAT3** | **STAT3** | **STAT3** | **PTEN** | | **CDK4** | | **CCND1** |
| 5 | **CDK6** | **RAC1** | **FGF2** | **CDK4** | **CDK4** | **CREB1** | | **CDK6** | | **GRB2** |
| 6 | CCNE1 | **VEGFA** | **CREB1** | **CDK6** | **VEGFA** | **CDK4** | | **CREB1** | | **VEGFA** |
| 7 | **CCND2** | GRB2 | **VEGFA** | **CCND1** | **GRB2** | **GRB2** | | **GRB2** | | APP |
| 8 | CCNE2 | ESR1 | **GRB2** | **VEGFA** | FGF2 | ESR1 | | CCND2 | | **CDK4** |
| 9 | **RAC1** | **FGF2** | RAC1 | **GRB2** | **CREB1** | FGF2 | | **VEGFA** | | ESR1 |
| 10 | CDC25A | RAC1 | IRS1 | **CREB1** | IRS1 | CCND2 | | **RAC1** | | **CDK6** |

**Supplementary Table S2.** Summary of ROC parameters. AUC – Area Under Curve, CI – confidence interval (95%), S – Sensitivity and Sp – specificity. S and Sp are given for optimal cut-off points, calculated by the Youden index method.

| **DE miRNAs** | **AUC (%)** | **Lower Border CI (%)** | **Upper Border**  **CI (%)** | **Cut-off point** | **S (%)** | **Sp (%)** |
| --- | --- | --- | --- | --- | --- | --- |
| **miR-1246** | 92.3 | 86.1 | 98.6 | 92.5 | 80.6 | 94.1 |
| **miR-150-5p** | 87.2 | 78.8 | 95.5 | 31.1 | 75 | 85.3 |
| **miR-4454+miR-7975** | 85.6 | 76.3 | 94.9 | 74.2 | 75 | 88.2 |
| **miR-142-3p** | 83.3 | 73.8 | 92.9 | 168.1 | 86.1 | 76.5 |
| **miR-15a-5p** | 82.4 | 72.4 | 92.5 | 27.8 | 86.1 | 70.6 |
| **miR-144-3p** | 82.2 | 72.4 | 92 | 53.0 | 55.6 | 97.1 |
| **miR-126-3p** | 81.0 | 71.0 | 91.1 | 106.8 | 75 | 79.4 |
| **miR-15b-5p** | 80.1 | 70.0 | 90.1 | 44.1 | 83.3 | 64.7 |
| **miR-191-5p** | 79.6 | 69.1 | 90.1 | 27.2 | 55.6 | 97.1 |
| **miR-4516** | 78.4 | 67.7 | 89.2 | 88.4 | 55.6 | 88.2 |
| **miR-630** | 78.2 | 67.3 | 89.1 | 149.8 | 80.6 | 64.7 |
| **miR-106b-5p** | 75.0 | 63.3 | 86.7 | 23.4 | 47.2 | 97.1 |

**Supplementary Table S3.** Results of attribute selection algorithms InfoGainAttributeSelection and Correlation Feature and Correlation Feature Selection.

| InfoGainAttributeEval | | | |
| --- | --- | --- | --- |
| Attribute | Average merit | | Average rank |
| miR-1246 | 0.598 ± 0.022 | | 1 ± 0,14 |
| miR-144-3p | 0.401 ± 0.038 | | 2 ± 0.24 |
| miR-150-5p | 0.335 ± 0.02 | | 3.4 ± 0.85 |
| miR-15a-5p | 0.309 ± 0.02 | | 4.2 ± 0.69 |
| miR-142-3p | 0.306 ± 0.018 | | 4.6 ± 0.67 |
| miR-106b-5p | 0.273 ± 0.019 | | 6.3 ± 0.72 |
| miR-191-5p | 0.266 ± 0.018 | | 7.2 ± 0.86 |
| miR-4516 | 0.246 ± 0.018 | | 8 ± 1.17 |
| miR-15b-5p | 0.242 ± 0.029 | | 9 ± 1.31 |
| miR-4454+miR-7975 | 0.236 ± 0.017 | | 9.3 ± 0.96 |
| miR-126-3p | 0.162 ± 0.072 | | 11.2 ± 0.4 |
| miR-630 | 0.054 ± 0.091 | | 11.8 ± 0.4 |
| CfsSubsetEval | | | |
| Attribute | | Numbers of fold (%) | |
| miR-1246 | | 50 (100%) | |
| miR-144-3p | | 50 (100%) | |
| miR-150-5p | | 50 (100%) | |
| miR-15a-5p | | 34 (68%) | |
| miR-142-3p | | 8 (16%) | |
| miR-106b-5p | | 13 (26%) | |
| miR-191-5p | | 6 (12%) | |
| miR-4516 | | 50 (100%) | |
| miR-15b-5p | | 15 (30%) | |
| miR-4454+miR-7975 | | 50 (100%) | |
| miR-126-3p | | 2 (4%) | |
| miR-630 | | 1 (2%) | |

**Supplementary Table S4.** Variables in developed classification models 1 and 2 with different panels of miRNAs; a0 – constant, a_1_ – coefficient of predictor x_1_, a_2_ – coefficient of predictor x_2_.

| **Coefficient** | **Constant a_0_** | **Coefficient for predictor x_1_ (a_1_)** | **Coefficient for predictor x_2_ (a_2_)** |
| --- | --- | --- | --- |
| x1 = miR-1246  x2 = miR-150-5p | 4.4117 | 0.07091 | -0.31985 |
| x1 = miR-1246  x2 = miR-144-3p | -0.94138 | 0.03202 | -0.02179 |

**Supplementary Table S5.** Quality parameters for diagnostic classification Models 1 and 2 in training and test sets for expression measurements by NanoString method.

| **Name** | **Model 1** | | **Model 2** | |
| --- | --- | --- | --- | --- |
|  | **miR-1246**  **miR-150-5p** | **miR-1246**  **miR-150-5p** | **miR-1246**  **miR-144-3p** | **miR-1246**  **miR-144-3p** |
| Set | Training | Test | Training | Test |
| Area under Curve (AUC) | 98.6% | 100% | 93.9% | 95.2% |
| Confidence Interval (CI) lower limit | 96.4% | - | 85.8% | 85.2% |
| CI upper limit | 100% | - | 100% | 100% |
| Cut-off point (Youden index) | 0.44 | 0.44 | 0.56 | 0.56 |
| Sensitivity | 96.4% | 100% | 92.9% | 92.3% |
| Specificity | 95.2% | 92.3% | 95.2% | 87.5% |
| Coefficient of determination (R2) | 0.74 | - | 0.66 | - |
| Root Mean Square Error (RMSE) | 0.23 | - | 0.29 | - |

**Supplementary Table S6.** Table of confusion in the set for Models 1 and 2. TN – true negatives; TP – true negatives; FN – false negatives; FP – false positives

|  |  | **Actual state** | | | |
| --- | --- | --- | --- | --- | --- |
|  |  | **miR-1246**  **miR-150-5p** | | **miR-1246**  **miR-144-3p** | |
|  |  | 0 | 1 | 0 | 1 |
| **Prediction** | 0 | 12 (TN) | 0 (FN) | 12 (TN) | 1 (FN) |
|  | 1 | 1 (FP) | 8 (TP) | 1 (FP) | 7 (TP) |

**Supplementary Table S7.** Variables in developed classification Model 3 for RT-qPCR data; a0 – constant, a_1_ – coefficient of predictor x_1_, a_2_ – coefficient of predictor x_2_.

| **Coefficient** | **Constant a_0_** | **Coefficient for predictor x_1_ (a_1_)** | **Coefficient for predictor x_2_ (a_2_)** |
| --- | --- | --- | --- |
| x1 = miR-1246  x2 = miR-150-5p | 55.160 | -1.616 | 4.277 |

**Supplementary Table S8.** Quality parameters for diagnostic classification Model 3 in training and test sets for expression measurements by RT-qPCR data.

| **Name** | **miR-1246**  **miR-150-5p** | **miR-1246**  **miR-150-5p** |
| --- | --- | --- |
| Set | Training | Testing |
| Area under Curve (AUC) | 99.7% | 94.6% |
| Confidence Interval (CI) lower limit | 99.0% | 83.9% |
| CI upper limit | 100% | 100% |
| Cut-off point (Youden index) | 0.2 | - |
| Sensitivity | 96.4% | 100% |
| Specificity | 94.1% | 91.7% |

**Supplementary Table S9.** Table of confusion in the set for Model 3. TN – true negatives; TP – true negatives; FN – false negatives; FP – false positives

|  |  | **Actual state** | |
| --- | --- | --- | --- |
|  |  | **miR-1246**  **miR-150-5p** | |
|  |  | 0 | 1 |
| **Prediction** | 0 | 11 (TN) | 0 (FN) |
|  | 1 | 1 (FP) | 14 (TP) |

**Supplementary Table S10.** Variables in developed classification Model 4 for external microarray dataset; a0 – constant, a_1_ – coefficient of predictor x_1_, a_2_ – coefficient of predictor x_2_.

| **Coefficient** | **Constant a_0_** | **Coefficient for predictor x_1_ (a_1_)** | **Coefficient for predictor x_2_ (a_2_)** |
| --- | --- | --- | --- |
| x1 = miR-1246  x2 = miR-150-5p | -2.57007 | 0.05412 | 0.49059 |

**Supplementary Table S11.** Table of confusion in the set for Model 4. TN – true negatives; TP – true positives; FN – false negatives; FP – false positives

|  |  | **Actual state** | |
| --- | --- | --- | --- |
|  |  | **miR-1246**  **miR-150-5p** | |
|  |  | 0 | 1 |
| **Prediction** | 0 | 182 (TN) | 37 (FN) |
|  | 1 | 42 (FP) | 187 (TP) |

**Supplementary Table S12.** List of primers for RT-qPCR.

| Primers | Sequence (5’ to 3’) |
| --- | --- |
| miR-144-3p | UACAGUAUAGAUGAUGUACU |
| miR-142-3p | UGUAGUGUUUCCUACUUUAUGGA |
| miR-150-5p | UCUCCCAACCCUUGUACCAAGUG |
| miR-15a-5p | UAGCAGCACAUAAUGGUUUGUG |
| miR-15b-5p | UAGCAGCACAUCAUGGUUUACA |
| miR-126-3p | UCGUACCGUGAGUAAUAAUGCG |
| miR-4454 | GGAUCCGAGUCACGGCACCA |
| miR-7975 | AUCCUAGUCACGGCACCA |
| miR-1246 | AAUUGGAUUUUUGGAGCAGG |
| miR-191-5p | CAACGGAAUCCCAAAAGCAGCUG |
| miR-4516 | GGGAGAAGGGUCGGGGC |
| miR-630 | AGUAUUCUGUACCAGGGAAGGU |
| miR-106b-5p | UAAAGUGCUGACAGUGCAGAU |
| miR-103-3p | AGCAGCAUUGUACAGGGCUAUGA |
| miR-199b-5p | CCCAGUGUUUAGACUAUCUGUUC |
