# Supplementary material for: Identification of serum miR-1246 and miR-150-5p as novel diagnostic biomarkers for high-grade serous ovarian cancer
Source: Sci Rep. 2023 Nov 7;13:19287. doi: 10.1038/s41598-023-45317-7 (PMC10630404; doi:10.1038/s41598-023-45317-7)
Supplement: Supplementary file 8 — Supplementary Legends. [file 41598_2023_45317_MOESM8_ESM.docx]

**Figure S1** Zero-order protein-protein interaction (PPI) network of DE miRNAs target genes. Data was drawn using NetworkAnalyst 3.0 platform. Node size reflects the number of protein-protein interactions, with larger nodes representing a greater number of annotated interactions.

**Figure S2** Analysis of pathway over-representation (A) GO - Biological process (BP) and (B) KEGG pathway analyses within 1024 DE miRNAs target genes. All functional annotations were performed with the hypergeometric test and Bonferroni adjustment (corrected P value (FDR) ≤ 0.05); KEGG - Kyoto Encyclopedia of Genes and Genomes.

**Figure S3** (A) IPA canonical pathway map for “Molecular mechanisms of cancer” displaying DE miRNAs target genes. Purple boxes represent proteins involved in the common network. Sollid arrows, known interactions; dotted arrows, indirect interactions; inverted triangle, kinase; double circle, complex; filled shapes, genes from our list; (B) KEGG pathway map for “PI3K-Akt signalling pathway” displaying DE miRNAs target genes. The genes that red coded are targeted by DE miRNAs.

**Figure S4** Weighted Gene Co-expression Network Analysis (WGCNA). (A) Cluster analysis of samples of HGSOC to detect outliers (white-to-red linear gradient colour associated with a corresponding clinical variable, grey when missing data); (B) Determination of soft-thresholding power in weighted gene co-expression network analysis (WGCNA); (C) Hierarchical clustering dendrograms of identified co-expressed miRNAs in modules in HGSCO serum samples. Each coloured row represents a colour-coded module which contains a group of highly connected genes. A total of five modules were identified; (D) A scatterplot of miRNAs significance for FIGO stage versus module membership in the yellow module. miRNAs significance and module membership exhibit a very significant correlation, implying that hub miRNAs of the yellow module also tend to be highly correlated with the FIGO stage.

**Figure S5** Identification of modules using WGCNA analysis associated with the clinical traits of HGSOC. (A) Heatmap of the correlation between module eigengenes and clinical traits of HGSOC; (B) Venn diagram of the overlap between yellow module genes and DE miRNAs; (C) KEGG pathway and WikiPathways analysis of miRNA target genes from the yellow module. All functional annotations were performed with the hypergeometric test and Bonferroni adjustment (corrected P value (FDR) ≤ 0.05). KEGG – Kyoto Encyclopedia of Genes and Genomes.

**Figure S6** ROC curves and AUC for DE miRNAs were obtained based on data on the expression level of miRNA molecules using the NanoString platform.
